# Hepatocellular carcinoma-linked *AXIN1* mutations drive low Wnt/β-catenin activity enabling niche-independent growth and YAP/TAZ signaling

**DOI:** 10.1016/j.isci.2025.114501

**Published:** 2025-12-20

**Authors:** Anton J. Venhuizen, Yvanka van Os, Milo L. Kaptein, Marleen T. Aarts, Despina Xanthakis, Ingrid Jordens, Madelon M. Maurice

**Affiliations:** 1 Oncode Institute and Center for Molecular Medicine, University Medical Center Utrecht, 3584 CX Utrecht, the Netherlands

**Keywords:** Molecular interaction, Molecular network, Cancer

## Abstract

AXIN1 organizes assembly of a destruction complex that degrades the transcriptional co-activator β-catenin, thereby preventing inappropriate Wnt/β-catenin signaling. In hepatocellular carcinoma (HCC), *AXIN1* mutations associate with a poor-prognosis subtype distinct from β-catenin-mutant HCC (*CTNNB1*). To assess how *AXIN1*-deficiency drives HCC, we introduced HCC-associated *AXIN1* and *CTNNB1* mutations in human liver cancer cells and liver-derived organoids. We show that *AXIN1* mutations activate Wnt/β-catenin signaling to varying but generally lower levels than *CTNNB1* mutations. Strikingly, premature 5′-end stop codons do not yield knockout mutations but drive alternative translation of N-terminally truncated AXIN1 variants with partial suppressor activity. All *AXIN1* variants enable liver progenitor organoids to grow without Wnt and R-spondin, indicating downstream Wnt/β-catenin activation. Additionally, Wnt/β-catenin signaling inversely correlates with YAP/TAZ-mediated signaling, leaving higher YAP/TAZ activity in *AXIN1*-mutant versus *CTNNB1*-mutant cells. Thus, *AXIN1* mutations drive physiologically relevant Wnt/β-catenin activation, providing a permissive environment for YAP/TAZ signaling, thereby distinguishing them from *CTNNB1* mutations.

## Highlights

• *AXIN1* and *CTNNB1* mutations drive distinct levels of Wnt/β-catenin activation
• *AXIN1* mutations confer Wnt- and RSPO-independent growth of liver organoids
• Wnt signaling inversely correlates with YAP/TAZ activity in liver cancer cells
• *AXIN1* mutations allow for simultaneous activity of Wnt/β-catenin and YAP/TAZ pathways

## Graphical abstract

**Figure:**
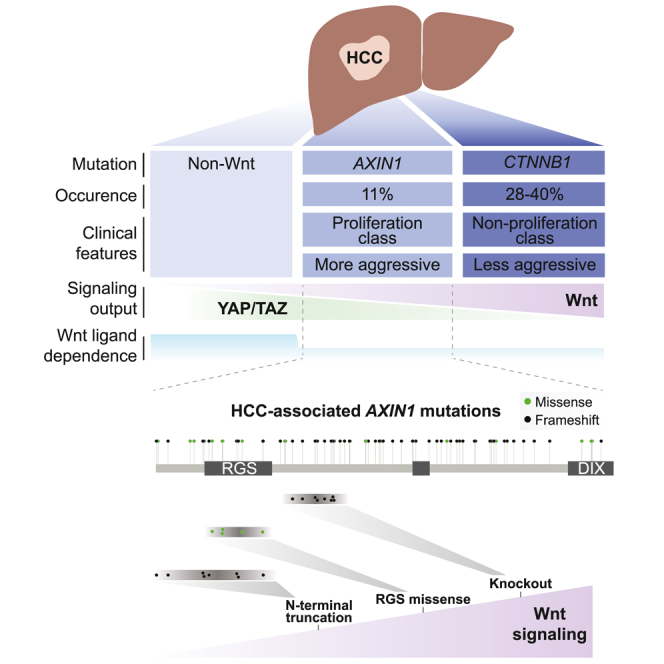

## Introduction

Hepatocellular carcinoma (HCC) is the most prevalent form of liver cancer and the third deadliest malignancy worldwide, accounting for over 800.000 deaths per year.^1^ Although HCC is a heterogeneous disease, mutational alterations that affect core components of the Wnt/β-catenin pathway are identified in a prominent fraction of patients.^2^ In the healthy liver, the evolutionary conserved Wnt/β-catenin pathway governs development, homeostasis, injury-induced regeneration, and zonation.^3^ Furthermore, levels of Wnt/β-catenin signaling determine metabolic identity and correlate with proliferation rates of hepatocytes that constitute the majority of liver parenchyma cells.^4^ The primary outcome of Wnt/β-catenin pathway activation is stabilization of the transcriptional co-factor β-catenin, which promotes its nuclear entry and complex formation with T cell factor/lymphoid enhancer factor (TCF/LEF) to mediate the transcription of Wnt target genes.^5^ In the absence of Wnts, cytosolic β-catenin levels are constitutively targeted for proteolysis by a multiprotein β-catenin destruction complex. AXIN1, the principal coordinator of this complex, brings together the scaffold APC and kinases GSK3 and CK1 to orchestrate efficient phosphorylation, ubiquitination and proteasomal degradation of β-catenin.^6^ Upon binding of Wnts to their receptors at the cell surface, the destruction complex is inactivated, resulting in β-catenin accumulation and the activation of gene programs linked to stem cell maintenance and proliferation.^5^

For HCC, the most frequent alterations in Wnt pathway components comprise activating mutations in β-catenin (gene name: *CTNNB1*, mutated in 28%–40% of cases) and inactivating mutations in *AXIN1* (mutated in 11% of cases).^7^ Historically, *AXIN1* and *CTNNB1* mutations in HCC were considered functionally equivalent, as cell lines harboring mutations in either gene displayed increased complex formation between β-catenin and TCF/LEF, indicative of Wnt pathway activation.^8^ Recent studies, however, linked *CTNNB1* and *AXIN1* mutations to molecularly distinct HCC subtypes, each with divergent pathological implications. *CTNNB1* mutations generally result in a Wnt-high expression profile and are predominantly found in the class of “non-proliferative” tumors that associate with better prognosis.^9^ By contrast, *AXIN1* mutations are linked to the “proliferative” Wnt-low class of more aggressive HCC tumors, hallmarked by poor differentiation. To what extent *AXIN1* mutations activate Wnt signaling in HCC, remains a highly debated issue. *Axin1*-deficient mice were reported to develop liver tumors without indications of Wnt pathway activation.^10^ Instead, these tumors displayed elevated levels of YAP/TAZ signaling, NOTCH pathway activation and fetal gene expression. In line with these findings, a recent study showed that YAP/TAZ-mediated signaling is indispensable for liver tumor formation in *Axin1*-deficient mice.^11^ Moreover, overexpression of YAP promotes expansion of progenitor-like murine hepatocytes, further linking YAP/TAZ signaling to the “proliferative”, dedifferentiated HCC subtype associated with *AXIN1* mutations.^12^

In contrast to these reports, we and others established a connection between *AXIN1* mutations and elevated Wnt signaling. For instance, HCC tumors harboring *AXIN1* mutations displayed upregulation of a subset of Wnt target genes.^13^^,^^14^ Furthermore, several cancer-associated *AXIN1* missense mutations in the N-terminal RGS domain of AXIN1 were found to induce Wnt signaling and drive hyperplastic growth in *Drosophila* wing discs.^15^ Recent studies further showed that various *AXIN1* missense and truncating mutations promote Wnt/β-catenin pathway activation and, moreover, signaling levels directly correlated with tumorigenic potential in the liver.^16^^,^^17^

In summary, *AXIN1*-mutant liver tumors depend on both β-catenin and YAP/TAZ signaling; however, the relative contributions of these pathways remain disputed. While *AXIN1*-mutant HCC cell lines are generally considered Wnt-low compared to *CTNNB1*-mutant cells, a comprehensive comparison between both mutation types in HCC cells with a similar genetic background is lacking.^18^ Furthermore, it is unclear whether *AXIN1* mutations endow niche factor-independent growth in the liver, as observed for other Wnt pathway mutations in various tissues, such as the colon.^19^

To address these issues, we introduced a variety of HCC-associated *AXIN1* mutations as well as activating *CTNNB1* mutations in the liver cancer cell line Huh7, HEK293T cells, and mouse liver progenitor organoids. We observe that missense and truncating *AXIN1* mutations activate Wnt/β-catenin signaling, although to varying degrees and less pronounced than *CTNNB1* mutations. Unexpectedly, we found that the insertion of premature stop codons in the 5′ exonic regions of *AXIN1* do not result in knockout but rather promote alternative translation of N-terminally truncated AXIN1 variants that display partially retained suppressor activity. Mouse liver progenitor organoids harboring *Axin1* alterations could be propagated in the absence of Wnt and R-spondin (RSPO) ligands, indicating that *Axin1* mutations enable niche-independent Wnt pathway activation in the liver. Notably, we uncover an inverse correlation between the degree of mutation-induced Wnt pathway activation and the capacity of cells to activate YAP/TAZ signaling. Our findings thus provide an explanation for why Wnt-high, *CTNNB1*-mutant tumors generally lack YAP/TAZ activity while Wnt-low, *AXIN1*-mutant tumors associate with enhanced YAP/TAZ-mediated gene transcription and a more clinically aggressive phenotype.

## Results

### Missense mutations within the RGS domain of AXIN1 drive Wnt/β-catenin signaling

**Figure 1:**
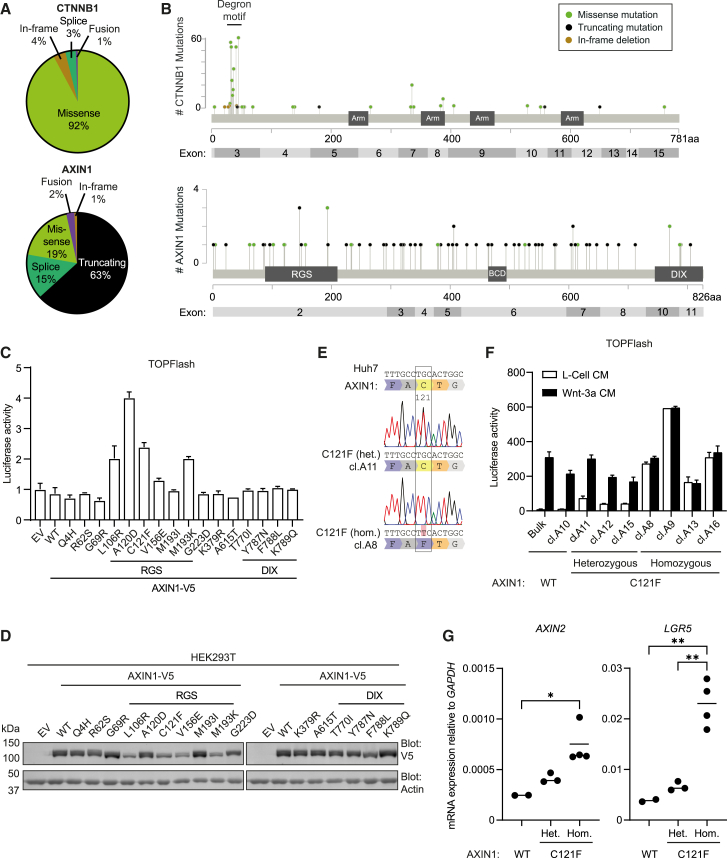
Missense mutations within the RGS domain of AXIN1 drive Wnt/β-catenin signaling (A) Query of liver cancer data deposited by MERiC, INSERM, MSK, AMC, RIKEN, and TCGA, containing sequencing data from a total of 1,233 patient samples. (B) Schematic depicting the number of mutations per amino acid in *CTNNB1* and *AXIN1*, based on the same liver cancer databases as used in (A). Color-coding depicts the type of mutation. Arm, Armadillo repeats; BCD, β-catenin binding domain. (C) TOPFlash reporter assay of overexpressed AXIN1 variants in HEK293T cells. EV indicates empty vector control. Graph shows a representative experiment (*n* = 3) with mean ± SD of *n* = 2 independent wells. Mutations in RGS or DIX domain are indicated. (D) Western blot of AXIN1-V5 expression levels of assay shown in (C). Actin was used as loading control. (E) Sanger sequencing of Huh7 clones harboring a heterozygous or homozygous base substitution in *AXIN1* exon 2, resulting in the C121F mutation. For all sequencing data, see Figure S4. (F) TOPFlash reporter assay comparing non-modified Huh7 cells to clones harboring heterozygous and homozygous AXIN1 C121F mutations. Cells were treated with Wnt-3a CM or L-Cell CM as control. Graph shows a representative experiment (*n* = 3) with mean ± SD of *n* = 2 independent wells. (G) RT-qPCR experiments depicting expression of Wnt target genes *AXIN2* and *LGR5* relative to the household gene *GAPDH*. Each dot represents the mean of three biological replicates per clone. The horizontal line represents the mean of all tested clones with the indicated genotype. Significance was determined using one-way ANOVA analysis. ∗ indicates *p* ≤ 0.05, ∗∗ indicates *p* ≤ 0.01. Non-significant comparisons were left out for clarity.

To assess the roles of *CTNNB1* and *AXIN1* mutations in HCC, we examined mutational patterns in these genes as listed in the cBioPortal database (Figures 1A and 1B).^20^^,^^21^^,^^22^^,^^23^^,^^24^^,^^25^^,^^26^ For *CTNNB1*, the majority of missense mutations (88%) locate to the N-terminal degron motif of β-catenin and thus interfere with its phosphorylation, ubiquitination, and proteasomal degradation.^27^ By contrast, genetic alterations in *AXIN1* are primarily truncating and missense mutations that are scattered throughout the gene. We previously demonstrated that cancer-associated single amino acid substitutions within the RGS domain of AXIN1 promote unfolding and the formation of soluble nano-aggregates, leading to impaired AXIN1 polymerization, loss of APC binding, and reduced destruction complex activity.^15^ We set out to assess the functional consequences of all *AXIN1* missense variants reported in the TCGA HCC database, using a β-catenin-dependent luciferase reporter assay (TOPFlash) in HEK293T cells.^28^ Most AXIN1 variants carrying a mutant RGS domain induced ligand-independent activation of the Wnt/β-catenin pathway upon overexpression (Figure 1C). Concomitantly, these mutations disrupted AXIN1 polymerization and stability, as shown by loss of cytosolic puncta formation (Figure S1A) and diminished overall protein expression levels (Figure 1D), in line with the previously reported L106R variant.^15^ AXIN1 A120D formed the only exception, as for this variant β-catenin-mediated reporter activity was induced while puncta formation and protein levels remained unaltered (Figures 1D and S1A), in line with its decreased propensity for aggregation in comparison with the other RGS missense variants (Figure S1B).^15^ The AXIN1 A120D mutation likely interferes with APC binding directly, given its position in the APC interaction interface (Figure S1C).^17^ Notably, introduction of missense mutations in the AXIN1 DIX domain did not lead to ligand-independent activation of Wnt/β-catenin signaling in this model system, even though polymerization was perturbed (Figures 1C, S1A, and S1D). In a previous study, we showed that such variants display impaired suppressor activity when expressed at lower, endogenous concentrations,^29^ indicating that these more subtle defects may be overcome by increased AXIN1 concentrations due to overexpression.

To evaluate the functional consequences of RGS cancer mutations at the endogenous level, we introduced homozygous AXIN C121F and L106R mutations in HEK293T cells using prime editing and CRISPR/Cas9-assisted knockin, respectively (Figures S2A and S2C). All clones harboring these HCC-associated mutations displayed hyperactivation of Wnt/β-catenin signaling in the absence of Wnt supplementation (Figures S2B and S2D). Furthermore, we confirmed loss of interaction between AXIN1 L106R and APC in these clones (Figures S2E and S2F), substantiating the previously observed loss of structural integrity of the RGS domain.^15^ Together, these data suggest that cancer-associated AXIN1 RGS mutations promote Wnt/β-catenin signaling by disrupting destruction complex assembly and function.

Next, we examined how AXIN1 RGS missense cancer mutations impact Wnt/β-catenin signaling in a liver cancer background, using Huh7 cells. This cell line carries no mutations in core components of the Wnt/β-catenin pathway,^30^ and is responsive to treatment with Wnt-3a and RSPO1, indicating that the Wnt/β-catenin signaling route is intact (Figure S3). Furthermore, Huh7 cells were not sensitive to treatment with the Wnt secretion inhibitor LGK974, demonstrating that these cells do not depend on Wnt ligands for their growth and survival (Figure S3). We employed prime editing to introduce AXIN1 C121F mutations in Huh7 cells (Figures 1E and S4A).^31^ We obtained heterozygous and homozygous mutant clones, for which activation of Wnt/β-catenin signaling in the absence of Wnt ligand directly correlated with their mutant allele frequency (Figure 1F). In line with these findings, expression of the Wnt target genes *AXIN2* and *LGR5* was elevated in a stepwise manner in heterozygous and homozygous AXIN1 C121F clones (Figure 1G). Together, these data provide compelling evidence that AXIN1 RGS missense mutations induce Wnt/β-catenin pathway activation in liver cancer cells.

### Frameshift mutations in 5′ coding regions yield an N-terminally truncated AXIN1 variant with partially retained functionality

**Figure 2:**
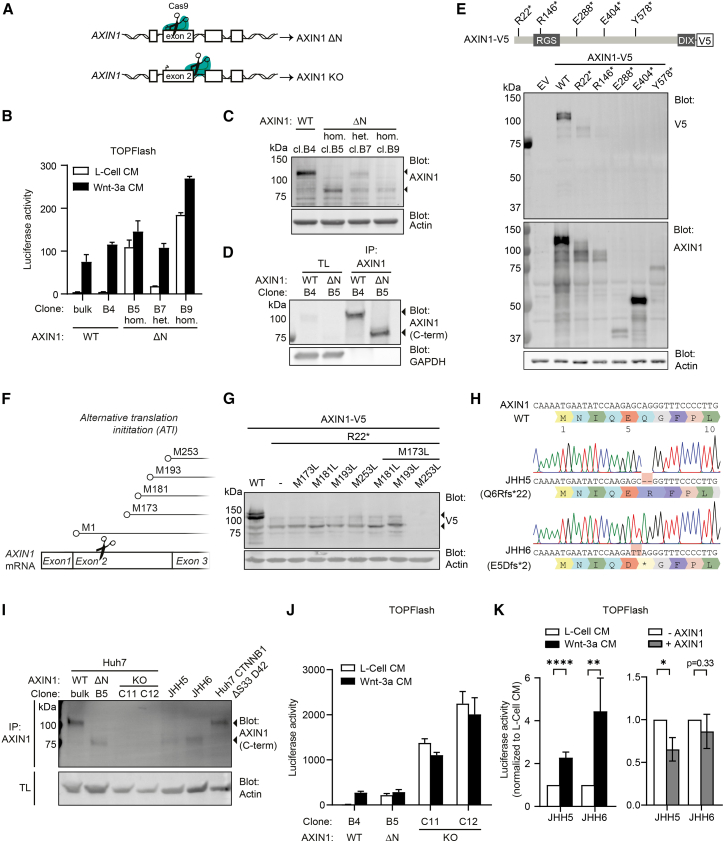
Frameshift mutations in 5′ coding regions yield an N-terminally truncated AXIN1 variant with partially retained functionality (A) Schematic depicting the different single guide RNA recognition sites used to generate CRISPR/Cas9-mediated frameshifts in the *AXIN1* locus. Targeting close to the translation initiation site results in an N-terminally truncated (ΔN) AXIN1 variant, whereas targeting in the 3′ region of exon 2 results in a full AXIN1 knockout (KO). (B) TOPFlash reporter assay comparing non-modified Huh7 cells to clones with heterozygous and homozygous frameshift mutations early in the *AXIN1* gene. Huh7 cells were treated with Wnt-3a CM or L-Cell CM as control. Graph shows a representative experiment (*n* = 3) with mean ± SD of *n* = 2 independent wells. (C) Western blot of endogenous AXIN1 levels in Huh7 clones harboring *AXIN1* frameshift mutations close to the translation start site. Arrows highlight the AXIN1 WT and ΔN variants. Actin was used as loading control. Western blot shown is representative of 2 independent experiments. (D) Western blot of immunoprecipitated AXIN1 from Huh7 WT clone B4 and AXIN1 ΔN clone B5. Immunoprecipitation was performed to enrich for AXIN1 for visualization purpose. Western blot shown is representative of 3 independent experiments. (E) Top: Schematic depicting AXIN1 with a C-terminal V5 tag. Lines indicate the sites at which stop codons are introduced. Termination mutations are all derived from the HCC databases described in Figure 1B. Bottom: Western blot of HEK293T lysates overexpressing AXIN1-V5 truncated variants stained for V5 and AXIN1. Actin was used as loading control. EV, empty control vector. Western blot shown is representative of 2 independent experiments. (F) Schematic depicting candidate start sites for recognition by the translation initiation machinery upstream in the *AXIN1* locus. The 4 methionines found in the 3′ part of exon 2 are the main candidates for alternative translation initiation. (G) Western blot of HEK293T cells overexpressing AXIN1 variants harboring truncating mutation R22∗ in combination with different methionine-to-leucine substitutions (similar side chain size), stained for V5. Actin was used as loading control. Western blot shown is representative of 2 independent experiments. The upper and lower arrowheads indicate the full-length and truncated AXIN1 variants, respectively. (H) Sanger sequencing of HCC cell lines JHH5 and JHH6, both harboring 5′ frameshift mutations in *AXIN1*. (I) Western blot of immunoprecipitated AXIN1 (using ab AF3287) from JHH5, JHH6, and different *AXIN1*-mutated Huh7 cell lines. For AXIN1 detection, ab C76H11 was used. Actin was used as loading control. Western blot shown is representative of 3 independent experiments. The upper and lower arrowheads indicate the full-length and truncated AXIN1 variants, respectively. (J) TOPFlash reporter assay comparing non-modified Huh7 cells to clones with different AXIN1 truncations. Cells were treated with Wnt-3a CM or L-Cell CM as control. Graph shows a representative experiment (*n* = 3) with mean ± SD of *n* = 2 independent wells. (K) TOPFlash reporter assay of JHH5 and JHH6 non-modified (left) and AXIN1 overexpressing (right) cells. Cells were treated with L-Cell CM or Wnt-3a CM (left), or only L-Cell CM (right). Graphs show the mean of three biological replicates normalized to L-Cell CM ± SD. Significance was determined using one-way ANOVA. ∗ indicates *p* ≤ 0.05, ∗∗ indicates *p* ≤ 0.01, ∗∗∗∗ indicates *p* ≤ 0.0001.

The majority of *AXIN1* mutations in HCC are frameshift mutations that, in case nonsense mediated decay is inefficient, may drive formation of truncated AXIN1 variants (Figure 1A). Frameshift mutations that locate to 5′ regions of the coding parts of the gene, are predicted to lead to an AXIN1 deletion phenotype, either due to nonsense-mediated decay or loss of most functional domains in case of truncation.^17^^,^^32^ In line with this assumption, Cre-mediated excision of *AXIN1* exon 2, encoding for amino acids 1 to 292, was used previously to generate conditional *AXIN1* knockout mice.^14^ We next aimed to assess the functional consequences of frameshift mutations in *AXIN1* close to the translation start site by using CRISPR/Cas9-mediated gene editing in Huh7 cells (Figures 2A and S4B).^33^ In line with loss of AXIN1 suppressor function, these mutations induced basal Wnt/β-catenin signaling and the effects correlated with *AXIN1* mutant allele frequency (Figure 2B). Unexpectedly, however, Western blotting revealed that these Huh7 clones retained expression of an AXIN1 fragment with a lower molecular weight, as shown by an antibody recognizing the central region of AXIN1 (AF3287) (Figure 2C, lower arrow). This AXIN1 variant could also be detected using an antibody directed against the AXIN1 C-terminus (C76H11), indicating this concerned an N-terminally truncated variant (Figure 2D, lower arrow). In corroboration, an overexpression construct encoding for mutation R22∗ also resulted in an AXIN1 variant with reduced molecular weight, which was detectable using a C-terminal V5 epitope tag, both on western blot and by immunofluorescence (Figures 2E and S5A). Furthermore, these N-terminally truncated AXIN1 variants maintained their capacity to form cytosolic puncta, confirming that their C-terminal DIX domain remained intact (Figure S5A). Truncations resulting from frameshift mutations downstream of AXIN1 position R146 could not be visualized via the C-terminal V5 tag, while these variants were still detected by the AF3287 antibody, recognizing the central part of AXIN1 (Figures 2E and S5A). Thus, while stop codons downstream of R146 generate C-terminally truncated AXIN1 variants, introduction of stop codons in the 5′ region of *AXIN1* exon 2 result in AXIN1 truncations that lack the N-terminus (ΔN) but still harbor the C-terminal DIX domain. Based on these findings, we hypothesized that ΔN AXIN1 variants may be generated by alternative translation initiation.^34^ To examine this assumption, we eliminated potential alternative start sites by mutating methionines M173, M181, M193 and M253 in exon 2 to leucine within AXIN1 R22∗ variants (Figure 2F). Indeed, combined mutation of M173 and M253 disrupted expression of the AXIN1 R22∗ variant, indicating that these start sites are utilized for alternative translation initiation (Figure 2G).

To test whether alternative translation initiation is physiologically relevant in the context of liver cancer, we evaluated AXIN1 expression in the HCC cell lines JHH5 and JHH6 that both harbor 5′ truncating mutations in exon 2 (Q6Rfs∗22 and E5Dfs∗2, respectively) (Figure 2H). Indeed, these cell lines expressed an AXIN1 variant at a similar molecular weight as our CRISPR/Cas9-modified Huh7 cells with 5′ truncations in *AXIN1* exon 2 (Figure 2I). Importantly, due to these alternative translation initiation sites, a robust AXIN1 knockout (KO) line would require truncating mutations downstream of the initiation sites M173 and M253. Indeed, introduction of frameshift mutations immediately downstream of M253 led to loss of detectable AXIN1 expression (Figures 2I and S4C). Of note, Huh7 AXIN1 KO cells displayed strongly increased levels of basal Wnt/β-catenin pathway activity in comparison to Huh7 AXIN1 ΔN cells (Figure 2J). Moreover, AXIN1 KO HEK293T cells also displayed increased Wnt/β-catenin signaling compared to AXIN1 ΔN HEK293T cells (Figures S5B–S5D). In agreement, JHH5 and JHH6, expressing N-terminally truncated AXIN1, still partially respond to Wnt3a stimulation and their basal Wnt/β-catenin pathway activity can be reduced by overexpressing exogenous AXIN1 (Figures 2K and S5E). Together, these results reveal a previously overlooked class of *AXIN1* 5′ frameshift mutations that drive expression of an N-terminally truncated protein with partially retained functionality.

### *AXIN1*-mutant HCC cells display moderate Wnt/β-catenin signaling, while *CTNNB1*-mutant cells are Wnt-high

**Figure 3:**
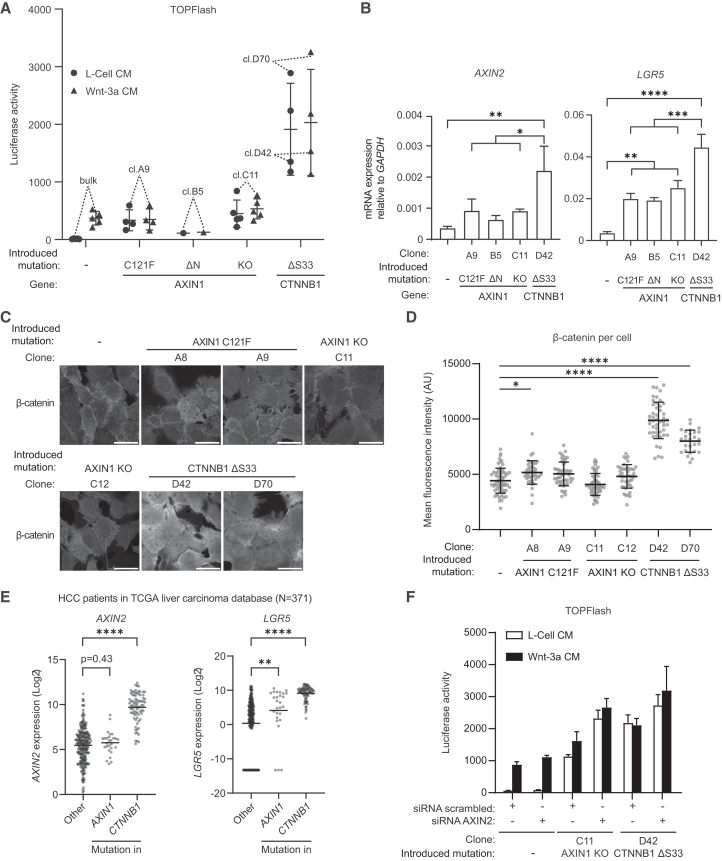
*AXIN1*-mutant HCC cells display moderate Wnt signaling levels, while *CTNNB1*-mutant cells are Wnt-high (A) TOPFlash reporter assay comparing non-modified Huh7 cells to clones carrying different *AXIN1* and *CTNNB1* mutations. Graph shows the mean ± SD for all tested clones of a representative experiment (*n* = 3), where one dot represents the mean of technical duplicates of one clone. The horizontal line represents the mean ± SD of all tested clones with the indicated genotype. Cells were treated with Wnt-3a CM or L-Cell CM as control. (B) RT-qPCR depicting expression of Wnt target genes *AXIN2* and *LGR5* relative to the household gene *GAPDH*. Graph shows the mean of three biological replicates per clone ± SD. Significance was determined using one-way ANOVA. (C) Immunofluorescence images of Huh7 lines harboring different Wnt pathway mutations, labeled for β-catenin. Scale bars represent 30 μm. (D) Quantification of immunofluorescence images belonging to the experiment in (C). Quantified *n* = 325 cells using an automated ImageJ script for which details are described in the “STAR Methods” section. Graph shows mean ± SD. Significance was determined using one-way ANOVA. (E) Analysis of expression data from the TCGA liver carcinoma database, accessed using the R2 analysis platform.^36^ Since log_2_ values cannot equal 0, all missing values in this dataset have been replaced with 0.0001, of which the log_2_ equals −13.2877. The horizontal line indicates the mean. Significance was determined using one-way ANOVA. (F) TOPFlash reporter assay comparing non-modified Huh7 cells to clones harboring *AXIN1* and *CTNNB1* mutations after siRNA-mediated knock-down of *AXIN2*. Graph shows a representative experiment (*n* = 3) with mean ± SD of *n* = 2 independent wells. ∗ indicates *p* ≤ 0.05, ∗∗ indicates *p* ≤ 0.01, ∗∗∗ indicates *p* ≤ 0.001, ∗∗∗∗ indicates *p* ≤ 0.0001. Non-significant comparisons were left out for clarity.

While activating *CTNNB1* mutations have been associated with Wnt/β-catenin hyperactivation in HCC, *AXIN1* mutations were linked to low or even completely absent Wnt/β-catenin signaling.^10^^,^^13^ To directly compare both mutation types in Huh7 cells, we introduced an in-frame deletion of β-catenin degron residue S33 using prime editing (Figure S4D).^35^ Clones carrying homozygous β-catenin ΔS33 mutations displayed higher Wnt/β-catenin pathway activation compared to different classes of AXIN1-mutant clones (Figure 3A). At the transcriptional level, *CTNNB1*-mutant Huh7 cells displayed increased expression of Wnt target genes *AXIN2* and *LGR5* (Figure 3B). In line with the luciferase reporter data, these genes were upregulated to a lower extent in *AXIN1*-mutant lines (Figure 3B). In further agreement, β-catenin levels were more strongly stabilized in *CTNNB1*-mutant versus WT and *AXIN1*-mutant Huh7 cells (Figures 3C and 3D). No significant difference in β-catenin accumulation between WT and *AXIN1*-mutated Huh7 clones was observed, corroborating the notion that β-catenin is an insensitive marker for Wnt pathway activation.^17^ Next, we queried the TCGA liver carcinoma database to examine the expression of *AXIN2* and *LGR5* in HCC patient samples (Figure 3E). *AXIN2* expression was slightly (but not significantly) upregulated in *AXIN1*-mutant HCC, while this Wnt target gene was significantly elevated in *CTNNB1*-mutant HCC. In agreement with our observations in Huh7 cells, *LGR5* expression displayed a significant upregulation in *AXIN1*-mutant HCC and these effects were more pronounced in *CTNNB1*-mutant HCC samples (Figure 3E). Together, our results show that both *AXIN1*-mutant and *CTNNB1*-mutant HCC display elevated Wnt/β-catenin signaling, although levels of pathway activation are more moderate for *AXIN1*-mutant cells.

AXIN2 normally acts as safeguard that curbs Wnt signaling in the absence of AXIN1.^17^^,^^37^^,^^38^ By contrast, β-catenin mutants with a disrupted degron motif are insensitive to AXIN2 levels.^37^ This may explain the difference in Wnt pathway activity between *CTNNB1*- and *AXIN1*-mutant HCC. Indeed, *AXIN2* knock-down in *AXIN1*-mutant Huh7 cells resulted in an increase in Wnt/β-catenin pathway activity to similar levels as *CTNNB1*-mutant cells (Figure 3F). Similarly, CRISPR/Cas9-mediated inactivation of *AXIN2* in *AXIN1*-mutant HEK293T cells resulted in synergistic Wnt/β-catenin pathway activation (Figure S6). These data confirm that elevated levels of AXIN2 partially compensate for impaired activity of its paralog in *AXIN1*-mutant tumors.

### Wnt/β-catenin pathway activation leads to dose-dependent inhibition of YAP/TAZ signaling

**Figure 4:**
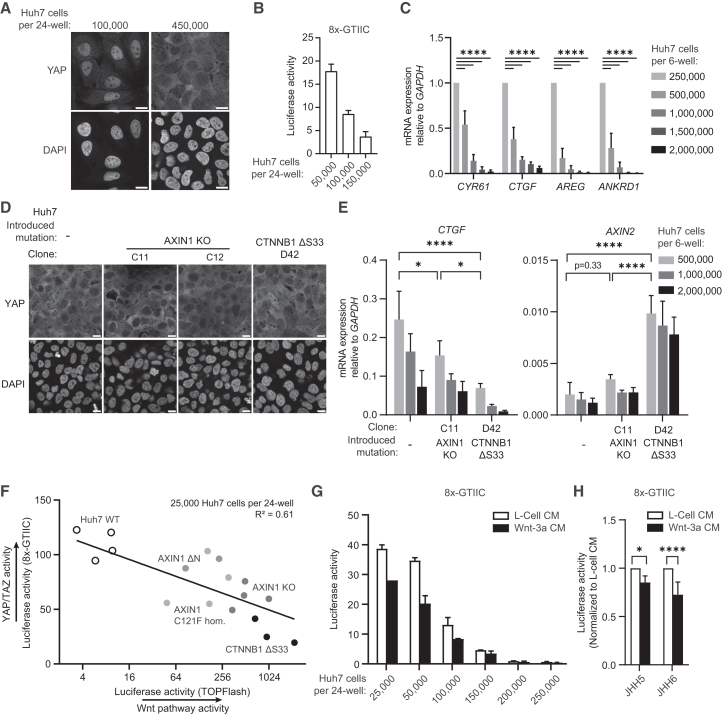
Wnt pathway activation leads to dose-dependent inhibition of YAP/TAZ signaling (A) Representative immunofluorescence images of Huh7 cells seeded at low and high cell density. Fixed cells were labeled for YAP and DAPI. Scale bars represent 30 μm. (B) 8xGTIIC reporter assay comparing Huh7 cells seeded at different cell densities. Graph shows a representative experiment (*n* = 2) with mean ± SD of *n* = 2 independent wells. (C) RT-qPCR depicting expression of YAP/TAZ target genes *CYR61*, *CTGF*, *AREG*, and *ANKRD1* relative to the household gene *GAPDH* in Huh7 cells seeded at different cell densities. Each condition was normalized to the condition with the lowest seeding density. Bars and error bars represent the mean of three biological replicates ± SD. Significance was determined using two-way ANOVA. (D) Representative immunofluorescence images of Huh7 clones with different genotypes seeded at 200,000 cells per 24-well. Fixed cells were labeled for YAP and DAPI. Scale bars represent 30 μm. (E) RT-qPCR depicting expression of YAP/TAZ target gene *CTGF* and Wnt target gene *AXIN2* relative to the household gene *GAPDH* for different Huh7 clones at increasing cell density. To directly compare all clones, relative quantification (RQ) values representing the expression compared to *GAPDH* were plotted. Bars and error bars represent the mean and standard deviation of three biological replicates. Significance was determined using two-way ANOVA. (F) Scatterplot comparing YAP-responsive reporter activity to TOPFlash reporter activity for a set of 17 Huh7 clones with different genotypes. Representative of *n* = 3 experiments. The coefficient of determination (R^2^) was determined using GraphPad analysis software. (G) 8xGTIIC luciferase assay comparing Huh7 cells seeded at different cell densities, either treated with Wnt-3a CM or L-Cell CM as control. Graph shows a representative experiment (*n* = 3) with mean ± SD of *n* = 2 independent wells. (H) 8x-GTIIC luciferase assay comparing JHH5 and JHH6 cells seeded at similar cell densities, either treated with Wnt-3a CM or L-Cell CM as control. Graph shows the mean of three biological replicates normalized to L-Cell CM ± SD. Significance was determined using one-way ANOVA. ∗ indicates *p* ≤ 0.05, ∗∗∗∗ indicates *p* ≤ 0.0001.

Previous studies have linked the occurrence of *AXIN1* mutations in HCC to inactivation of the Hippo signaling pathway and concomitant hyperactivity of YAP/TAZ-mediated transcription, although the underlying mechanisms have remained incompletely understood.^10^^,^^11^ To address this issue, we set out to compare the levels of YAP/TAZ signaling in Huh7 cells in the absence or presence of mutations in *AXIN1* and *CTNNB1*, as well as in JHH5/6 cells carrying an AXIN1 N-terminal deletion. First, we confirmed that these lines are dependent on Hippo signaling, as indicated by a dose-dependent reduction in YAP/TAZ activity upon treatment with the inhibitor MGH-C1 (Figures S7A and S7B). Since the Hippo signaling cascade is primarily regulated by mechanotransduction, we assessed whether this pathway is sensitive to alterations in Huh7 cell confluency.^39^ Compared to Huh7 cells at lower densities, cells that were growing near confluency displayed nuclear exclusion of YAP (Figure 4A), decreased YAP/TAZ-mediated luciferase reporter activity (Figure 4B) and downregulated expression of YAP/TAZ targets *CYR61*, *CTGF*, *AREG*, and *ANKRD1* (Figure 4C). These data indicate that the Hippo pathway is functional in Huh7 cells.

We next assessed whether Wnt/β-catenin pathway alterations affect YAP localization in Huh7 cells seeded at relatively high confluency. Nuclear YAP levels were not visibly altered in *AXIN1*-deficient Huh7 cells compared to non-modified control cells (Figures 4D and S7C). Moreover, both *AXIN1-* and *CTNNB1*-mutant cells displayed reduced levels of YAP/TAZ-mediated transcription when compared with control cells at varying seeding densities (Figures 4E and S7D). To perform a more comprehensive investigation of the relationship between Wnt/β-catenin pathway activity and Hippo signaling, we examined how varying levels of β-catenin-mediated transcription affect YAP/TAZ-dependent reporter activities in a large set of *AXIN1*- or *CTNNB1*-mutant Huh7 clones (Figure 4F). The results clearly revealed an inverse correlation between levels of Wnt/β-catenin pathway activation and YAP/TAZ transcriptional activity (Figure 4F). In an earlier study, the liver-specific Wnt target gene TBX3 was reported to repress YAP/TAZ signaling, providing a potential mechanism for the negative correlation between both pathways.^40^ We, however, did not detect significantly upregulated levels of TBX3 in either *AXIN1*- or *CTNNB1*-mutant cells, which may reflect either a selective advantage for clones with low expression of the anti-proliferative factor TBX3, or variability in TBX3 levels across HCC subtypes (Figure S7E).^40^

To further investigate how both pathways are interlinked, we treated control Huh7 cells and JHH5/6 cells with Wnt-3a conditioned medium (CM) (Figures 4G and 4H). Wnt-3a supplementation negatively regulated YAP/TAZ activation in a reporter assay, suggesting that there is crosstalk between both pathways that is not exclusive to *AXIN1*- or *CTNNB1*-mutant cells. Next, we used different small molecules to modulate Wnt/β-catenin and YAP pathway activity. Notably, treatment with the small molecule K-975, which inhibits TEAD transcription by blocking its interaction with YAP/TAZ, did not affect Wnt/β-catenin signaling in any of the Huh7-derived cell lines (Figures S7F–S7H), suggesting that alterations in YAP/TAZ-mediated transcription do not affect Wnt/β-catenin pathway activity. On the other hand, treatment with CHIR99021, a GSK3 inhibitor that activates Wnt/β-catenin signaling, downregulated expression of YAP target genes *CTGF*, *CYR61*, and *ANKRD1* in both non-modified and *AXIN1*-mutant Huh7 cells (Figures S7F–S7G). Importantly, similar effects of CHIR99021 treatment were observed in *CTNNB1*-mutant cells, indicating that GSK3 inhibition might act on YAP/TAZ signaling by a mechanism partially independent of β-catenin-dependent transcription (Figure S7H). Together, our results indicate that Wnt pathway activation negatively impacts transcription of YAP/TAZ-associated target genes in a dose-dependent manner in HCC cells.

### *Axin1* mutations promote Wnt ligand-independent growth in mouse liver progenitor organoids

**Figure 5:**
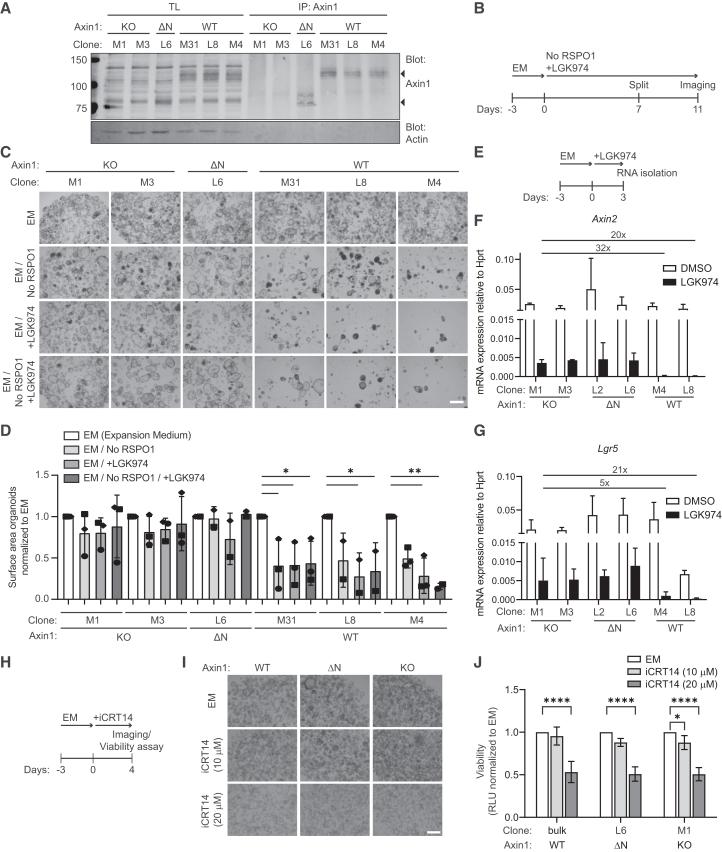
*Axin1* mutations promote Wnt ligand-independent growth in mouse liver progenitor organoids (A) Western blot of immunoprecipitated Axin1 from WT and *Axin1*-mutant mouse liver progenitor organoids. Actin was used as loading control. Western blot shown is representative of 2 independent experiments. (B) Diagram displaying the setup of the cell viability experiment performed in (C). (C) Representative brightfield images of mouse liver progenitor organoids cultured in expansion medium (EM) with and without 10% RSPO1 CM and 500 nM LGK974 of *n* = 2 experiments. Scale bar represents 500 μm. EM, expansion medium. (D) Quantification of biological replicates (*n* = 2/3) as performed in (C). Quantification was performed by determining cell surface area of the organoids using OrganoSeg analysis software.^42^ Each condition was normalized to organoids cultured in EM. EM, expansion medium. Graph shows mean ± SD. Significance was determined using one-way Anova. (E) Diagram of the experiment setup for the RT-qPCR depicting expression of (F) *Axin2* and (G) *Lgr5* relative to household gene *Hprt*. Organoids were treated with 500 nM LGK974 for 3 days. Bars and error bars represent the mean of three biological replicates ± SD. Fold-change differences between the indicated conditions were shown. (H) Diagram displaying the setup of the cell viability experiment performed in (I, J). (I) Representative brightfield images of liver progenitor organoids cultured in expansion medium with 10 or 20 μM iCRT14. Scale bar, 500 μm. EM, expansion medium. (J) Viability of WT and Axin1 mutant organoids cultured in EM supplemented with either 10 or 20 μM iCTR14. Graph shows the mean of four biological replicates normalized to EM ± SD. EM, expansion medium. Significance was determined using two-way ANOVA. ∗ indicates *p* ≤ 0.05, ∗∗ indicates *p* ≤ 0.01, ∗∗∗∗ indicates *p* ≤ 0.0001. Non-significant comparisons were left out for clarity.

Our results argue that *AXIN1* mutations drive Wnt/β-catenin signaling in HCC (Figure 3A). To determine whether these mutations suffice to acquire niche factor independence in a physiological setting, we introduced truncating Axin1 mutations in a mouse liver progenitor organoid line (Figures S8A and S8B).^41^ In line with our findings in human cells, the introduction of frameshift mutations in upstream 5′ regions (residue 15) led to the expression of N-terminally truncated Axin1 variants (Axin1 ΔN), whereas frameshift mutations introduced more downstream (residue 287) induced loss of detectable Axin1 protein (Axin1 KO) (Figures 5A and S8C). Proliferation and propagation of mouse liver progenitor organoids largely depends on Wnt/β-catenin pathway activation by RSPO1 supplementation and autocrine Wnt secretion.^41^ Strikingly, both Axin1 ΔN and Axin1 KO liver organoids displayed RSPO1-independent growth and were insensitive to treatment with the Wnt secretion inhibitor LGK974 (Figures 5B–5D, S8D, and S8E).^43^ Notably, we observed similar expansion rates for both Axin1 ΔN and Axin1 KO lines upon RSPO1 withdrawal and porcupine inhibitor treatment, suggesting that both classes of *Axin1* mutations can drive niche factor-independence in the liver (Figure 5D).

To determine how *Axin1* mutations influence Wnt/β-catenin pathway activity in these organoids, we assessed the effect of short-term LGK974 treatment on Wnt target gene transcription (Figures 5E–5G). Although treatment drastically reduced expression of *Lgr5* and *Axin2* in both WT and mutant organoid lines, residual expression of these Wnt target genes was approximately 5- to 40-fold times higher in both *Axin1*-mutant lines (Figures 5F and 5G). Of note, LGK974 treatment did not affect *Axin1* expression (Figure S8F). In addition, these organoids remain dependent on β-catenin dependent signaling, as treatment with the β-catenin-responsive transcription inhibitor iCRT14 significantly reduces the outgrowth of these organoids (Figures 5H–5J).^44^ Together, these results suggest that low levels of Wnt/β-catenin signaling are sufficient to sustain proliferation of liver progenitor cells.

**Figure 6:**
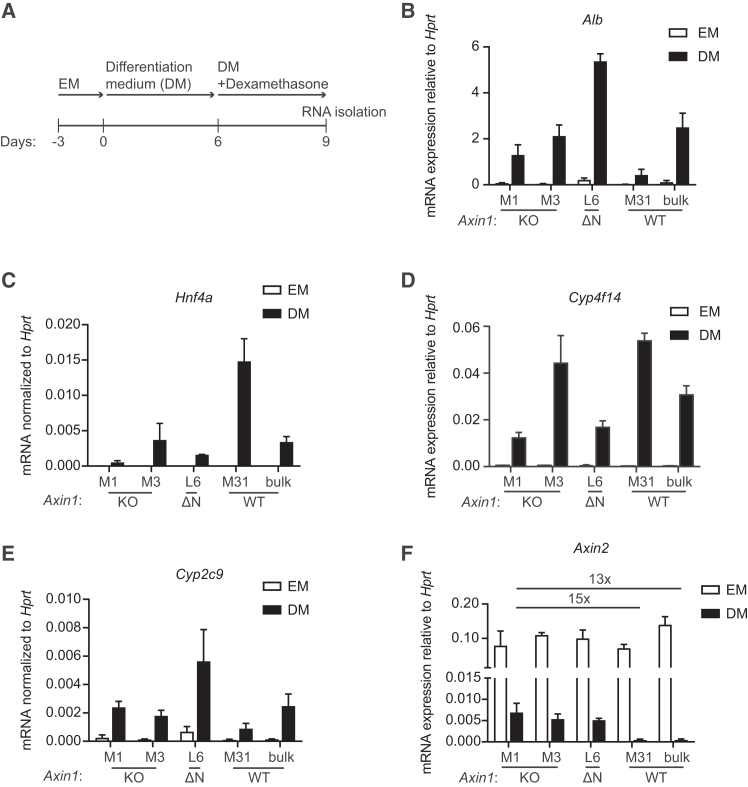
*Axin1* mutations promote Wnt/β-catenin pathway activity in mouse liver progenitor organoids with hepatocyte characteristics (A) Diagram displaying the differentiation protocol. (B–F) RT-qPCR experiments for (B) *Alb* (coding for Albumin), (C) *Hnf4a*, (D) *Cyp4f14*, (E) *Cyp2c9*, and (F) *Axin2* mRNA levels, respectively, relative to household gene *Hprt*. Fold-change differences between the indicated conditions were depicted. Bars and error bars represent the mean of three biological replicates ± SD. EM, expansion medium; DM, differentiation medium.

To determine the effects of *Axin1* mutations in organoids with hepatocyte features, we applied a previously defined differentiation protocol (Figure 6A).^41^ All organoid lines were capable of differentiating toward the hepatocyte lineage in these conditions, as evidenced by increased expression of *Alb*, encoding Albumin (Figure 6B), hepatocyte-associated transcription factor *Hnf4*^45^ (Figure 6C), and the hepatocyte-specific metabolic enzymes *Cyp2c9* and *Cyp4f14* (Figures 6D and 6E).^41^ Despite their induced differentiation, *Axin1*-mutant organoids retained a 15-fold increase in expression of the Wnt target gene *Axin2* when compared to WT organoid lines (Figure 6F). Together, these results indicate that *Axin1* mutations promote Wnt/β-catenin pathway activity in organoids with liver progenitor as well as hepatocyte characteristics.

## Discussion

Mutations in the Wnt/β-catenin pathway-associated genes *AXIN1* and *CTNNB1* are inherently linked to the development of HCC.^2^^,^^46^ Recent genome sequencing efforts of HCC patient cohorts revealed that mutations in *AXIN1* and *CTNNB1* are mutually exclusive and associate with distinct tumor characteristics, aggressiveness, and clinical prognosis.^9^ In this study, we aimed to examine the differences in downstream signaling events of each mutation type in HCC. Consistent with earlier studies, Wnt/β-catenin pathway activation was strongly induced upon introduction of β-catenin mutations in the liver cancer cell line Huh7.^13^^,^^47^^,^^48^ By contrast, introduction of *AXIN1* mutations induced only low levels of Wnt/β-catenin signaling, but with clear physiological relevance, as illustrated by the acquired Wnt/RSPO1-independent growth properties of *Axin1*-mutant mouse liver progenitor organoids. Importantly, our findings therefore challenge the claim that *AXIN1* mutations drive HCC development independent of the Wnt/β-catenin pathway.^10^ Notably, *Axin1*-mutant organoids that were treated with the Wnt secretion inhibitor LGK974 still displayed a reduction in Wnt target gene expression, suggesting that *AXIN1* mutations do not lead to complete uncoupling of the signaling cascade from the Wnt receptor complex. Thus, *AXIN1* mutations likely provide a low level of Wnt/β-catenin signaling that is sufficient for survival and proliferation and still allows for regulation by upstream signaling events, which is reminiscent of findings in MAPK signaling where residual levels of pathway activity were sufficient to evade apoptosis.^49^ In line with our findings, optimal Wnt signaling levels for HCC formation were estimated to be lower than those required for intestinal tumors, further supporting the view that *AXIN1* mutations facilitate “just-right” levels of Wnt signaling to enable HCC development.^50^^,^^51^

The majority of *AXIN1* mutations found in HCC are truncating and missense mutations. Although we and others have previously established a Wnt pathway-promoting role for missense mutations in the AXIN1 RGS domain, the physiological relevance of this mutation class in liver cancer has not been determined.^15^^,^^16^^,^^17^ Here, we confirm that RGS-associated AXIN1 missense mutations are capable of driving Wnt/β-catenin signaling in liver cancer cells. In addition, we found that truncating mutations near the canonical start site of *AXIN1* result in alternative translation initiation and, consequently, expression of an N-terminally truncated variant with residual tumor suppressor function. Notably, alternative translation initiation may represent a wider mechanism for tumor suppressors in cancer, considering a report claiming that generally more than 50% of CRISPR-cas9-mediated “knockout” cell lines retain expression of the targeted gene.^34^ As AXIN1 ΔN variants lack the tankyrase (TNKS)-binding and RGS domains, tumors harboring these mutations will be insensitive to TNKS1/2 inhibitors that stabilize AXIN1 or to the small molecule KYA1797, that binds AXIN1 RGS and increases β-catenin phosphorylation by promoting GSK3 activity.^52^^,^^53^ Instead, inhibition of SIAH1/2, ubiquitin ligases that regulate AXIN1 stability by associating with its more downstream GSK3 binding domain, might provide an effective strategy to stabilize AXIN1 ΔN cancer variants.^54^ Together, our findings underscore the need for careful evaluation of different cancer mutation types to improve patient stratification.^7^ In this context, emerging approaches such as circulating cell-free DNA (cfDNA)-based profiling, which enable the non-invasive detection of oncogenic mutations in HCC patients,^55^ may represent powerful tools to distinguish mutation classes and guide treatment strategies.

Several studies have linked *AXIN1* deficiency to increased signaling of the Hippo pathway effectors YAP and TAZ.^10^^,^^11^^,^^56^ Strikingly, however, none of the AXIN1 variants that we studied induced YAP/TAZ signaling in Huh7 cells, in agreement with a recent study.^16^ By contrast, elevated Wnt/β-catenin pathway activation attenuated YAP/TAZ signaling in a dose-dependent manner. These observations are in line with several studies that reported a negative correlation between both pathways in the liver.^57^^,^^58^ Our results suggest that the low levels of Wnt pathway activation induced by *AXIN1* mutations are sufficient to drive proliferation while, at the same time, providing a permissive environment for moderate levels of YAP/TAZ signaling. These effects of *AXIN1* mutations stand in contrast with the strong levels of Wnt/β-catenin pathway activation induced by *CTNNB1* mutations, which drive suppression of YAP/TAZ signaling. As mutations in core Wnt/β-catenin pathway components are late events in HCC development and *Axin1*-deficient mice show delayed tumorigenesis (>1 year),^10^^,^^14^^,^^59^ we anticipate that additional mutations are necessary for HCC progression and that differences in mutational background may impact on the degree of YAP/TAZ activation in these cancer cells. In addition, HCC is often associated with alterations in the tumor microenvironment, including stiffening of the extracellular matrix, which directly links to increased YAP/TAZ signaling.^60^ These factors might explain why transcriptional hyperactivation of YAP/TAZ in *Axin1*-deficient mouse tumors is not observed in our *AXIN1*-mutant cell cultures.^10^^,^^11^

In conclusion, our study reveals that different classes of *AXIN1* mutations endow liver cells with physiologically relevant Wnt/β-catenin signaling levels, although pathway activation is less pronounced than observed for *CTNNB1* mutations. Our results, however, indicate that the low levels of Wnt signaling induced in *Axin1*-mutant liver organoids are sufficient to sustain growth in the absence of Wnt ligands. Notably, inhibitors targeting the β-catenin/TCF interaction effectively suppressed growth, warranting further exploration of this class of drugs in liver cancer. In addition, we observed sensitivity to YAP/TAZ inhibition, irrespective of the presence of Wnt pathway mutations. Based on these findings, we propose that combination treatment with inhibitors for Wnt/β-catenin and YAP/TAZ signaling may offer a potential therapeutic avenue for HCC tumors harboring *AXIN1* mutations.^61^^,^^62^

### Limitations of the study

We acknowledge several limitations of our study. First, our models exhibit variable and complex genetic backgrounds, which may complicate the interpretation of signaling dependencies. Second, these systems do not recapitulate the tumor microenvironment, and thus cannot account for the contributions of stromal and immune components to Wnt/β-catenin and Hippo pathway regulation. Third, inhibition of β-catenin-mediated signaling by iCRT14 was assessed by organoid viability, rather than a direct reduction in target gene expression that was shown in earlier reports using other model systems.^44^^,^^63^^,^^64^^,^^65^ Fourth, as we did not employ genome-wide omics approaches, potential effects of AXIN1 mutations on additional signaling pathways might not have been captured.

## Resource availability

### Lead contact

Requests for information and resources should be directed to the lead contact, Madelon M. Maurice (m.m.maurice@umcutrecht.nl).

### Materials availability

Plasmids, cell lines, and organoid models presented in this article will be made available upon request.

### Data and code availability

• All data supporting the findings of this study are available within the manuscript and its supplemental information files. Original datasets generated during this study are available from the lead contact upon reasonable request.
• This paper does not report original code.
• Any additional information required to reanalyze the data reported in this paper is available from the lead contact upon request.

## Acknowledgments

We thank members of the laboratory of M.M.M. for discussions and suggestions. We thank Bart Spee (Utrecht University, Netherlands) for providing Huh7 cells, Ron Smits (Erasmus MC, Netherlands) for JHH5 and JHH6 cells, and Saskia van Mil (University Medical Center Utrecht, Netherlands) for mouse liver organoids. This work is part of the Oncode Institute, which is partly financed by the Dutch Cancer Society. This work was supported by the 10.13039/501100001826ZonMw TOP grant 91218050 and 10.13039/501100001826ZonMw Open competition grant 09120242410077 (to M.M.M.); 10.13039/501100004622Dutch Cancer Society grant 13112 (to M.M.M.); NWO Gravitation project IMAGINE! (to M.M.M.); and Dutch Cancer Society/TKI-Life Sciences and Health grant 2022-PPS-1/14853 (to M.M.M.).

## Author contributions

A.J.V., conceptualization, formal analysis, investigation, visualization, methodology, and writing – original draft, review, and editing; Y.v.O., conceptualization, formal analysis, investigation, visualization, and methodology; M.L.K., conceptualization, formal analysis, investigation, visualization, and methodology; M.T.A., conceptualization, formal analysis, investigation, visualization, and methodology; D.X., formal analysis, investigation, and methodology; I.J., conceptualization, formal analysis, investigation, visualization, methodology, and writing – original draft, review, and editing; M.M.M., formal analysis, supervision, funding acquisition, investigation, visualization, methodology, and writing – original draft, review, and editing.

## Declaration of interests

M.M.M. is cofounder and shareholder of Laigo Bio B.V. and is inventor on patents related to E3 ligase-mediated membrane protein degradation (PCT/EP2021/066696 and PCT/EP2021/055551).

## Declaration of generative AI and AI-assisted technologies in the writing process

During the preparation of this work, the authors used ChatGPT (OpenAI) to assist with grammar checks and improvements in writing clarity. The authors reviewed and edited all generated text and take full responsibility for the final content of the manuscript.

## STAR★Methods

### Key resources table

**Table:** 

REAGENT or RESOURCE	SOURCE	IDENTIFIER
**Antibodies**		

mouse anti-Actin	MP Biomedicals	#691001; RRID: AB_2920628
mouse anti-GAPDH	Calbiochem	#CB1001; RRID: AB_2107426
mouse anti-β catenin	BD Transduction Laboratories	#610153; RRID: AB_397554
rabbit anti-GSK3β	Cell Signaling	#9315; RRID: AB_490890
mouse anti-APC	Abcam	#ab58 ALi 12-28; RRID: AB_568441
mouse anti-V5	Genscript	#A01724; RRID: AB_2622216
rabbit anti-V5	Sigma	#V8137; RRID: AB_261889
goat anti-AXIN1	R&D systems	#AF3287; RRID: AB_2062418
rabbit anti-AXIN1	Cell signaling	#C76H11; RRID: AB_2274550
mouse anti-YAP	Santa Cruz	#sc-101199; RRID: AB_1131430
Goat anti-rabbit IrDye680	Licor	#926-68070; RRID: AB_10956588
Goat anti-rabbit IrDye800	Licor	#926-32211; RRID: AB_621843
Goat anti-rabbit Alexa 488	Invitrogen	A11034; RRID: AB_2576217
Goat anti-rabbit Alexa 568	Invitrogen	A11036; RRID: AB_10563566
Goat anti-mouse Alexa 488	Invitrogen	A11029; RRID: AB_2534088
Goat anti-mouse Alexa 568	Invitrogen	A11031; RRID: AB_144696

**Chemicals, peptides, and recombinant proteins**		

Matrigel	BD bioscience/Corning	356231
B27	Life Technologies	17504-001
N-acetylcysteine	Sigma-Aldrich	A9165 5 gr
Gastrin	Sigma-Aldrich	G9145
Recombinant mouse Egf	Peprotech	315-09
Recombinant mouse Fgf10	Peprotech	450-61
Recombinant mouse Hgf	Peprotech	315-23
Nicotinamide	Sigma-Aldrich	N0636 100gr
LGK974	Cayman chemicals	#14072
K-975	Selleckchem	E1329
iCRT14	Med Chem Express	HY-16665
CHIR99021	Tocris Bioscience	4423
DAPT	Calbiochem	565770
Dexamethason	Sigma-Aldrich	D1756
Y-27623	Selleckchem	S1049
Dispase	Gibco	17105-041
lipofectamine RNAiMAX	Thermo Fisher	13778030
FuGENE6	Promega	E2691
MGH-CP1	Selleckchem	S9735

**Critical commercial assays**		

Dual-Luciferase Reporter Assay System	Promega	E1960
CellTiter-Glo Luminescent Cell Viability Assay	Promega	G7571

**Experimental models: Cell lines**		

HEK293T	ATCC	RRID: CVCL_0063
Huh7 adult hepatocellular carcinoma cell line	Laboratory of Dr. Bart Spee	RRID: CVCL_0336
JHH5 adult hepatocellular carcinoma cell line	Laboratory of Dr. Ron Smits	RRID: CVCL_0364
JHH6 adult hepatocellular carcinoma cell line	Laboratory of Dr. Ron Smits	RRID: CVCL_2788

**Experimental models: Organisms/strains**		

C57BL/6 mouse liver progenitor organoids	Laboratory of Dr. Saskia van Mil, isolated as previously described by Huch et al.^41^	N/A

**Oligonucleotides**		

oligonucleotide sequences for CRISPR/Cas9, PCR and RT-qPCR	Table S1	
siRNA AXIN2	Dharmacon	L-008809-00-0005
siRNA scrambled	Dharmacon	D-001810-01-20

**Recombinant DNA**		

TOPFlash reporter	Described by Tauriello et al.^66^	
FOPFlash reporter	Described by Tauriello et al.^66^	
(TK)-Renilla plasmid	Described by Tauriello et al.^66^	
8x-GTIIC luciferase reporter	Laboratory of Martijn Gloerich, modified from Ota et al.^67^	N/A
pcDNA3.1+ plasmid containing V5-tagged human *AXIN1* isoform b	Described by Anvarian et al.^15^	N/A
pSpCas9(BB)-2A-Puro (PX459) V2.0	Laboratory of Feng Zhang^33^	Addgene: #62988
pU6-pegRNA-GG-acceptor	Laboratory of David Liu^31^	Addgene: #132777
phU6-gRNA plasmid	Laboratory of Charles Gersbach^68^	Addgene: #53188
PE2-P2A-puromycin resistance cassette	Laboratory of Susanne Lens, based on plasmid by the David Liu lab^31^	Modified from Addgene: #132775

**Software and algorithms**		

ImageJ macro for quantification of β-catenin levels	This paper	See Method S1
ImageJ	NIH	https://imagej.nih.gov
GraphPad Prism version 9.0	GraphPad Software	https://www.graphpad.com
R2: Genomics Analysis and Visualization Platform	Department of Oncogenomics, Academic Medical Center Amsterdam	http://r2.amc.nl
Illustrator	Adobe	https://www.adobe.com
cBioportal	Cerami et al.^26^	https://www.cbioportal.org/
Benchling	Benchling [Biology Software]. (2025).	https://benchling.com.
OrganoSeg	Borten et al.^42^	https://github.com/JanesLab/OrganoSeg

**Other**		

Luminometer Centro LB960	Berthold	N/A
LSM700 confocal microscope	Zeiss	N/A
EVOS M5000 Imaging System	Thermo Fisher	N/A

### Experimental model and study participation details

#### Cell culture

Human embryonic kidney (HEK293T) cells were cultured in Dulbecco’s modified Eagle’s medium with 4500 mg/L glucose (Sigma-Aldrich). Huh7 cells were cultured in Dulbecco’s modified Eagle’s medium with 1000 mg/L glucose (Sigma-Aldrich). JHH5 and JHH6 cells were cultured in William’s E medium (Gibco). All cell culture media were supplemented with 10% fetal bovine serum (GE Healthcare), 2 mM L-alanyl-L-glutamine (Sigma-Aldrich), 100 units/mL penicillin and 100 μg/mL streptomycin (Invitrogen). Cells were cultured in 5% CO2 at 37°C. Wnt-3a conditioned medium was obtained from mouse L-Cells stably expressing and secreting mouse Wnt-3a. L-Cell conditioned medium was obtained from control L-Cells. RSPO1 conditioned medium was produced using HEK293T cells stably transfected with human RSPO1-V5. Wnt surrogate conditioned medium was produced by conditioned medium of HEK293T cells transfected with Wnt surrogate (kind gift from Claudia Janda, Prinses Maxima Center, Netherlands)^69^ and added to cultures at 0.5-1%. For small-molecule inhibition, cells were treated with 5μM CHIR99021 (Tocris Bioscience), 10μM K-975 (Selleckchem), MGH-CP1 (Selleckchem; various concentrations) and 10 or 20 μM iCRT14 (Med Chem Express), unless other concentrations are indicated. Cell lines were not further authenticated after receipt and were regularly tested for mycoplasm.

#### Liver progenitor organoid cultures

C57BL/6 mouse liver progenitor organoids were obtained from the lab of S.W.C van Mil (University Medical Center Utrecht) and were isolated as previously described.^41^ No new animal experiments were performed as part of this study. The original derivation of these organoids was approved by the ethics committee of the University Medical Center Utrecht and was in accordance with European law (CCD number: AVD115002015259). For culturing, organoids were mixed with 40 μL matrigel (BD bioscience) per droplet and seeded in a 24-well plate and grown in expansion medium (EM). Expansion medium was based on Advanced DMEM F-12 (Gibco) supplemented with 100 units/mL penicillin and 100 μg/mL streptomycin (Invitrogen), 2 mM L-alanyl-L-glutamine (Sigma-Aldrich), 10 mM HEPES (Gibco), 1% B27 (Life Technologies), 1.25 μM N-acetylcysteine (Sigma-Aldrich), 10 nM gastrin (Sigma-Aldrich), 50 ng/mL mouse Egf (Peprotech), 10% RSPO1-V5 conditioned medium, 100 ng/mL mouse Fgf10 (Peprotech), 10 mM nicotinamide (Sigma-Aldrich) and 25 ng/mL mouse Hgf (Peprotech). Organoids were split in a 1:3-1:6 ratio once every week by mechanical disruption or dissociated using TrypLE Express (Gibco) for the viability assays. For differentiation, RSPO1-V5 condition medium, Hgf and nicotinamide were removed from the expansion medium, and instead A8301 (50 nM, Tocris) and DAPT (10 nM, Calbiochem) were added. The last 3 days of differentiation, dexamethason (30 μM, Sigma-Aldrich) was supplemented to the medium. For growth dependency assays, organoid lines were split 1:4 and directly cultured in EM, EM without RSPO1, EM supplemented with 500 nM LGK974 (Cayman chemicals), or EM without RSPO1 and supplemented with 500 nM LGK974. Organoids were split 1:4 after 7 days of culturing and were grown for 5 more days. Culture medium was replaced every other day. For short LGK974 treatment, organoid lines were split 1:4 and cultured for 3 days with or without supplementation of 500 nM LGK974. During growth dependency assays, brightfield images of organoids were taken using the EVOS M5000 Imaging System (Thermo Fisher).

The sex of the cell lines and organoid donors was not part of the experimental setup and was not considered a biological variable.

### Method details

#### Constructs

TOPFlash and FOPFlash luciferase reporter plasmids and thymidine kinase (TK)-Renilla were described previously.^66^ 8x-GTIIC luciferase reporter was a kind gift from the lab of Martijn Gloerich (University Medical Center Utrecht, Netherlands), which is modified from a previously described vector.^67^ For AXIN1 overexpression, human *AXIN1* isoform b was cloned into pcDNA3.1+ containing a C-terminal V5 tag. *AXIN1* mutations were introduced by site-directed mutagenesis using Q5 high-fidelity polymerase (New England Biolabs) using primers produced by Integrated DNA Technologies (see Table S1). The sgRNA CRISPR/Cas9 vector PX459 V2.0 was kindly provided by the lab of Feng Zhang (Addgene, #62988). Single guide RNAs (sgRNA) were cloned into the PX459 plasmid according to protocol^33^(See Table S1 for guide sequences including overhangs). Prime editing guide RNAs were cloned into the pU6-pegRNA-GG-Vector, kindly provided by the lab of David Liu (Addgene, #132777). Annealed pegRNA spacers, pegRNA extensions, and pegRNA scaffold sequences with appropriate overhangs were inserted into the pU6-pegRNA-GG-Vector using Golden Gate assembly using BsaI-HfV2 (New England Biolabs) and T4 DNA ligase (New England Biolabs).^31^ For PE3 cloning, phU6-gRNA vector^68^ (Addgene, #53188) was digested using BbsI (Thermo Fisher) and subsequently ligated by T7 DNA ligase (New England Biolabs). CTNNB1 ΔS33 pegRNA and PE3 constructs were a kind gift from the lab of Sabine Fuchs.^35^ A modified version of the PE2 plasmid by the lab of David Liu (Addgene, #132775) containing PE2 coupled via a P2A peptide to a puromycin resistance cassette was kindly provided by the lab of Susanne Lens (University Medical Center Utrecht, Netherlands).

#### CRISPR/Cas9 and prime editing of cell lines

Huh7 and HEK23T cells were seeded in 10 cm dishes at 25% cell confluency and transfected one day later. For conventional CRISPR/Cas9-mediated targeting, cells were transfected using FuGENE6 (Promega), according to the manufacturer’s protocol, with a DNA mixture of 6 μg PX459 plasmid containing sgRNA and 1 μg CMV-GFP plasmid to validate transfection efficiency. For prime editing, Huh7 cells were transfected using FuGENE6 (Promega), with a DNA mixture containing 7.8 μg PE2-P2A-Puromycin plasmid, 2.6 μg pegRNA plasmid, 0.84 μg nicking sgRNA plasmid and 1 μg CMV-GFP. For both prime editing and CRISPR/Cas9 targeting, puromycin selection was started after 2 days by the addition of 2 μg/mL puromycin and maintained for an additional two days. After outgrowth, genomic DNA was isolated from the bulk population using the QiaAmp DNA micro kit (Qiagen) according to protocol. Genotyping of the bulk population was performed by genomic PCR using primers in Table S1 with either GoTaq Flexi DNA polymerase (Promega), or Q5 polymerase (NEB). PCR products were isolated from gel and sent for Sanger sequencing at Macrogen Europe using primers from Table S1. Editing efficiency was predicted using the TIDE tool.^70^ After confirming successful editing, single cells clones were genotyped as described above.

#### CRISPR/Cas9 editing of organoids

PX459 plasmid harboring an sgRNA against mouse *Axin1* was purified from E. Coli using the EndoToxin Free Plasmid Maxi Kit (Qiagen). For electroporation, three confluent wells of organoids from a 24-well plate per condition were dissociated with TrypLE express (Gibco). A cell-DNA mixture was prepared by adding 15 μg Cas9 plasmid, 1 μg CMV-GFP plasmid to visualize electroporation efficiency, and up to 100 μl of BTX express buffer (BTX online) containing 10 μM Y-27623 (Selleckchem). The cell-DNA mixture was transferred to an electroporation cuvette (NEPA) and electroporated using a NEPA21 electroporator (NEPA GENE) with 2× poring pulse (voltage: 175 V, length: 5 ms, interval: 50 ms, polarity: +, decay rate 10) and 5× transfer pulse (voltage: 20 V, length: 50 ms, interval: 50 ms, polarity ±, decay rate 40). Afterwards, cells were incubated for 20 minutes at room temperature in 500 μL opti-MEM (Gibco) containing 10 μM Y-27623, centrifuged at 300 x g at 4°C and embedded in matrigel. Subsequently, organoids were cultured in EM supplemented with 0.5% Wnt surrogate conditioned medium and 10 μM Y-27623. For selection, 1 μg/μL puromycin (Sigma-Aldrich) was supplemented to the culture medium for 2 days, 2 days after electroporation. To determine the editing efficiency, the organoids were harvested by dissociating the matrigel using 1 mg/mL dispase (Gibco) for 20 minutes at 37°C. Genotyping was performed as described above using primers in Table S1 after which single clones were established by single organoid picking and expansion.

#### Antibodies

The following primary antibodies were used for immunoblotting (IB) and immunofluorescence (IF): mouse anti-Actin (MP Biomedicals, #691001), mouse anti-GAPDH (Calbiochem, CB1001), mouse anti-β catenin (BD Transduction Laboratories, #610153), rabbit anti-GSK3β (Cell Signaling, #9315), mouse anti-APC (Abcam, ab58), mouse anti-V5 (Genscript, A01724), rabbit anti-V5 (Sigma, V8137), goat anti-AXIN1 (R&D systems, AF3287), rabbit anti-AXIN1 (Cell signaling, #2087, C76H11) against C-terminus AXIN1, mouse anti-YAP (Santa Cruz, sc-101199). The following secondary antibodies were used for IB and IF: goat-anti-rabbit IrDye680 or IrDye800 (Licor), goat-anti-rabbit Alexa 488 or 568, goat anti-mouse Alexa 488 or 568.

#### TOPFlash and 8x-GTIIC reporter assays

For the β-catenin-dependent TOPFlash reporter assay, Huh7 cells were seeded at 100,000 cells per 24-well, and HEK293T and JHH5/6 cells at 50,000 cells per 24-well. After one day, Huh7 cells or JHH5/6 cells were transfected with a total amount of 200 ng DNA per well consisting of 30 ng of either TOPFlash or FOPFlash, 20 ng of TK-Renilla, and 150 ng empty vector or 100 ng AXIN1-V5 supplemented with 50 ng empty vector using FuGene6 (Promega), according to manufacturer’s protocol. HEK293T cells were transfected with a total amount of 250 ng DNA per well consisting of 30 ng of either TOPFlash or FOPFlash, 5 ng of TK-Renilla, 50 ng of AXIN1-V5 overexpression constructs and 165 ng empty vector. For the TEAD-dependent 8x-GTIIC luciferase reporter, cells were seeded at various cell densities and transfected with a total amount of 200 ng DNA per well consisting of 30 ng of 8x-GTIIC Luc reporter, 20 ng of thymidine kinase (TK)-Renilla, and 150 ng empty vector using FuGene6. If applicable, 50% Wnt-3a conditioned medium (CM) or L-Cell CM was added 6 hours after transfection. The next day, cells were lysed using 1x Passive lysis buffer (Promega) for 20 minutes at room temperature and 20 μL was transferred to a white costar 96-well plate. Firefly Luciferase and Renilla were measured on a Berthold Luminometer Centro LB960 using the Dual-Luciferase reporter kit (Promega). For siRNA-mediated knock-down, cells were transfected with siRNA to a final concentration of 25 nM using lipofectamine RNAiMAX (Thermo) two days prior to transfection with reporter constructs. siRNA AXIN2: Dharmacon L-008809-00-0005. siRNA scrambled: Dharmacon D-001810-01-20.

#### Immunofluorescence confocal microscopy

HEK293T cells were seeded in 24-wells onto glass coverslips that were coated with laminin (Sigma-Aldrich) (2.5 μg/mL in PBS) for one hour at 37°C. The next day, cells were transfected with 100 ng of AXIN1 construct and 150 ng empty vector per well. The following day, cells were fixed in 4% paraformaldehyde (PFA) in 50 mM sodium phosphate buffer, pH 7.4. After 30 minutes, the reaction was quenched by 50mM NH_4_Cl for 30 minutes at room temperature. Next, samples were blocked in blocking buffer containing 2% BSA and 0.1% saponin in PBS for 30 min at room temperature and afterwards incubated with primary antibody in blocking buffer for 1 hour at room temperature. Cells were washed three times with blocking buffer before the addition of secondary antibody and DAPI (Merck) for 45 minutes at room temperature. After three washing steps with blocking buffer, cells were washed with milliQ water and mounted in Prolong Gold (Invitrogen, P36391). Images were acquired with a Zeiss LSM700 confocal microscope and analyzed using ImageJ. Images were quantified using a custom macro for ImageJ, which is provided in Method S1. This macro sets a threshold for signal intensity, subtracts background, and creates cellular outlines based on nuclear localization. Output was manually filtered for incompletely quantified cells.

For detection of β-catenin and YAP in Huh7 and HEK293T, cells were seeded on coverslips and fixed, stained and mounted the following day as described above.

#### Immunoprecipitation

For AXIN1 co-immunoprecipitation in HEK293T clones, cells were seeded in five 15 cm dishes (Corning) and cultured until confluency. Cells were resuspended in 5 mL lysis buffer containing 50 mM Tris pH 7.5, 150 mM NaCl, 0.5% Triton X-100, 10% glycerol, 5 mM EDTA, 1 mM DTT, 50 mM sodium, 10 μg/mL aprotinin, 10 μg/mL leupeptin and 100 μM sodium vanadate. Cells were lysed for 1 hour at 4°C and subsequently centrifuged for 20 minutes at 21130 x g at 4°C. 45 μL of lysate was collected as total cell lysate. For AXIN1 IP, per sample 1 μg of goat anti-AXIN1 antibody (AF3287) was added, followed by incubation overnight while tumbling at 4°C. 20 μL of equilibrated protein G-coupled beads (Millipore) was added to each sample, followed by a 2-hour incubation at 4°C while tumbling. Beads were washed 3 times with lysis buffer, after which proteins were eluted in 2x Sample buffer (SDS, β-mercaptoethanol, glycerol, Tris pH 6.8, bromophenol blue) and boiled for 6 min. For AXIN1 immunoprecipitation in Huh7, JHH5 or JHH6 cells, the same protocol was applied as for HEK293T cells, except that cells were seeded in eight 15 cm dishes (Corning) and resuspended in 500 μL RIPA buffer containing 25 mM Tris pH 7.6, 150 mM NaCl, 1% NP40, 1% deoxycholate, 0,1% SDS supplemented with 1 mM DTT and 50 mM sodium fluoride, aprotinin (10 μg/mL), leupeptin (10 μg/mL) and sodium vanadate (100 μM).

#### Western blotting

Proteins were loaded on SDS-PAGE gels and separated on molecular weight. After separation, proteins were transferred to a polyvinylidene fluoride (PVDF) membrane (Immobilon-F) (Millipore). After membrane blocking by Odyssey Blocking Buffer (Li-Cor) diluted 1:1 in Tris-buffered saline (TBS), membranes were incubated with primary antibody overnight at 4°C in TBS containing 0.05% Tween (TBS-T). After washing with TBS-T, membranes were incubated with secondary antibody for 60 min at RT in TBS-T. After additional washing steps with TBS-T and a final wash with TBS, membranes were scanned by a Typhoon Biomolecular Imager (GE Healthcare). Full blots of each experiment are shown in Figure S9.

#### Quantitative real-time PCR

Huh7 cells and organoids were lysed in the dish by addition of 350 μL RLT buffer containing 1:100 β-mercaptoethanol. RNA isolation was performed following the RNeasy Kit (Qiagen) protocol, with an additional step to remove genomic DNA by the RNase-free DNase set (Qiagen). Next, cDNA was made from RNA using the iScriptTM cDNA synthesis kit (Biorad). qRT-PCR was performed on a Biorad CFX96 using iQ SYBR Green (Biorad) with primer concentrations of 0.4 nM.

#### Viability assay

For Huh7 and JHH5/6 cells, cells with similar densities were seeded in 96-wells plates and treated with increasing concentrations of MGH-CP1 (Selleckchem) for 5 days. At day 5, cell viability was assessed by a CellTiter-Glo assay (Promega) according to manufacturer’s protocol.

For mouse liver progenitor organoids, single cells were seeded into a 48-well plate and grown in Expansion medium (EM) supplemented with either DMSO, 10 μM iCRT14 or 20 μM iCRT14 for 4 days. Images were taken at day 4 on an EVOS microscope. Cell viability was assessed by a CellTiter-Glo assay (Promega) according to manufacturer’s protocol.

### Quantification and statistical analysis

All statistical analyses were performed using GraphPad Prism version 9. Data are reported as mean ± SD. The statistical tests used, exact n values, and what n represents (biological clones, independent wells, or organoid cultures) are provided in the figure legends. Luciferase reporter assays were normalized to Renilla luciferase and analyzed using ANOVA-based tests as specified in the legends. RT-qPCR data were normalized to GAPDH (human) or Hprt (mouse) using the ΔΔCt method, and analyzed with one- or two-way ANOVA, as reported in the legends. Quantification of immunofluorescence (e.g., β-catenin, YAP/TAZ) was performed using ImageJ (see Method S1). Organoid growth and viability measurements were analyzed using one- or two-way ANOVA to assess effects of genotype and treatment as indicated in the figure legends. Significance is indicated as ∗ p ≤ 0.05, ∗∗ p ≤ 0.01, ∗∗∗ p ≤ 0.001, ∗∗∗∗ p ≤ 0.0001. Non-significant comparisons were left out for clarity.

## Supplemental information

Document S1. Figures S1–S9, Table S1, and Method S1
