# Supplementary material for: Hepatocellular carcinoma-linked AXIN1 mutations drive low Wnt/β-catenin activity enabling niche-independent growth and YAP/TAZ signaling
Source: iScience. 2025 Dec 20;29(1):114501. doi: 10.1016/j.isci.2025.114501 (PMC12811488; doi:10.1016/j.isci.2025.114501)
Supplement: Document S1. Figures S1–S9, Table S1, and Method S1 [file mmc1.pdf]

iScience, Volume *29*

Supplemental information

Hepatocellular carcinoma-linked *AXIN1* mutations drive low Wnt/β-catenin activity enabling niche-independent growth and YAP/TAZ signaling

Anton J. Venhuizen, Yvanka van Os, Milo L. Kaptein, Marleen T. Aarts, Despina Xanthakis, Ingrid Jordens, and Madelon M. Maurice

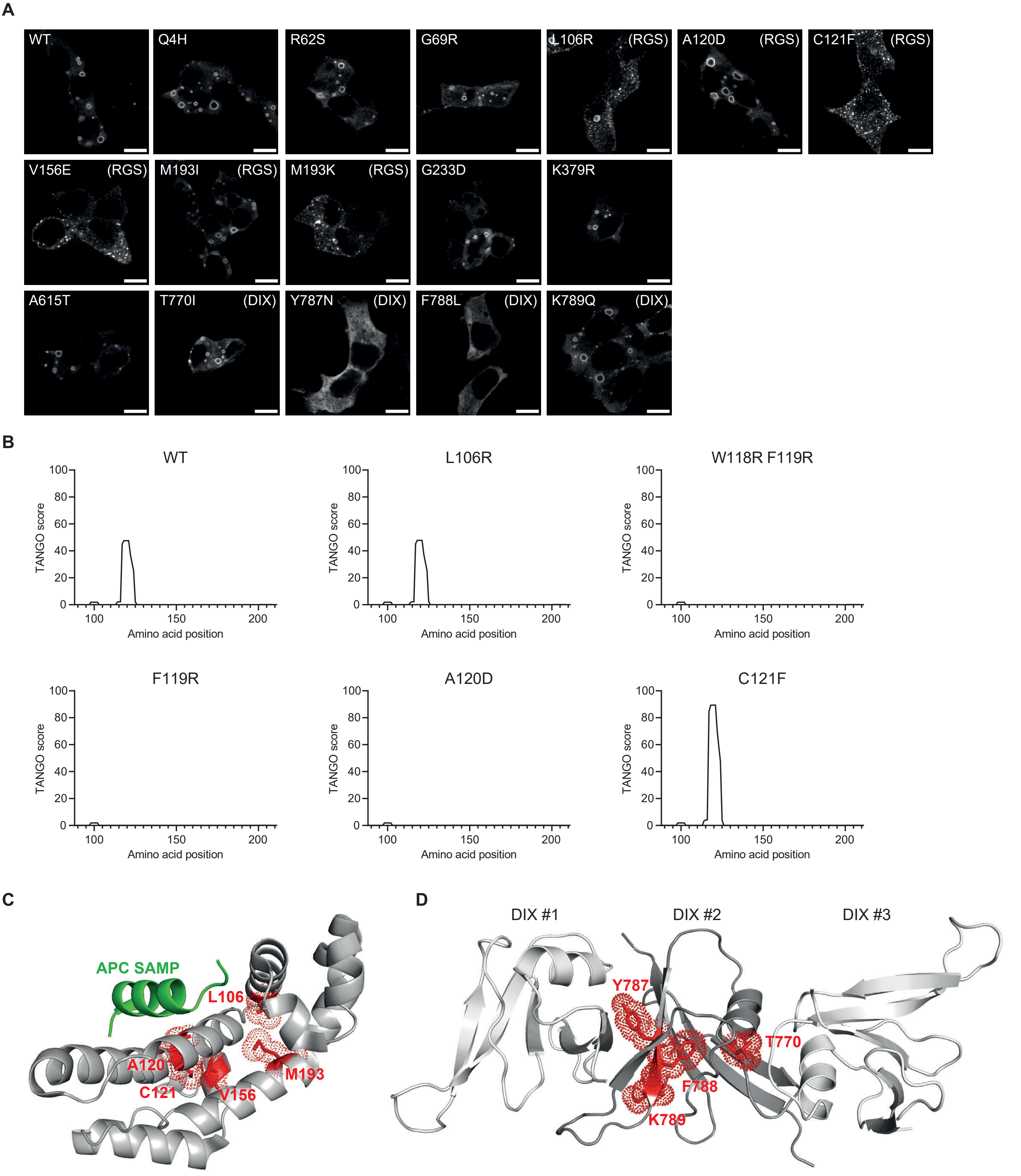

Supplemental figure S1. Related to figure 1 - Missense mutations within the RGS domain of AXIN1 drive Wnt signaling. (A) Representative immunofluorescence images of HEK293T cells overexpressing the indicated AXIN1-V5 variants. Fixed cells were labeled for V5. Scale bar represents 15 μm. **(B)** Aggregation propensity of indicated AXIN1 RGS variants predicted by TANGO. Cancer mutation A120D is predicted to function as rescue mutation for RGS aggregation, correlating with the observed puncta formation of this variant in (A). Cancer mutation C121F increases the aggregation propensity of the RGS domain, explaining why this cancer mutation shows stronger Wnt pathway activation than the L106R mutation. **(C)** Superimposition of residues mutated (red) in HCC onto the crystal structure of rat AXIN1 RGS (pdb: 1EMU). AXIN1-binding SAMP domain of APC is depicted in green. **(D)** Superimposition of residues mutated (red) in HCC onto the crystal structure of 3 polymerized rat AXIN1 DIX domains (pdb: 1WSP).

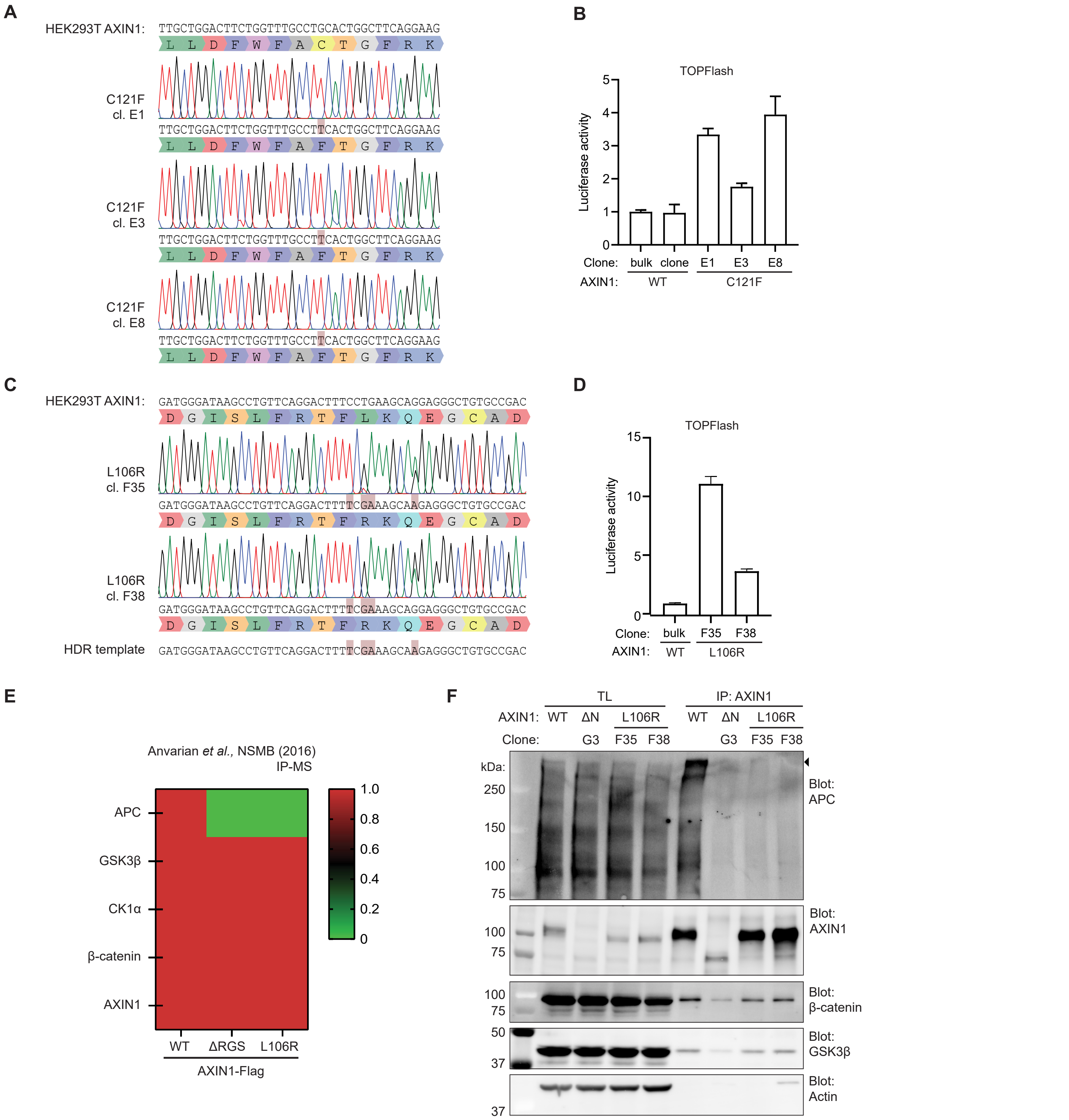

Supplemental figure S2. Related to figure 1 - Missense mutations within the RGS domain of AXIN1 drive Wnt/β-catenin signaling in HEK293T cells. (A) Sanger sequencing results of HEK293T clones harboring the AXIN1 C121F mutation introduced by prime editing. **(B)** β-catenin-dependent TOPFlash assay of HEK293T clones harboring the C121F mutation. The non-modified clone underwent the same procedure, but did not acquire the mutation. Graph shows a representative experiment (N=3) with mean +/- SD of N=2 wells. **(C)** Sanger sequencing results of HEK293T clones harboring the AXIN1 L106R mutation introduced via homologous recombination. **(D)** TOPFlash assay of HEK293T clones harboring the L106R mutation. Graph shows a representative experiment (N=3) with mean +/- SD of N=2 wells. **(E)** Heatmap displaying the relative amounts of β-catenin destruction complex members bound by FLAG-tagged AXIN1 mutants, as previously published15. **(F)** Co-immunoprecipitation experiment of endogenous AXIN1 (using AF3287 antibody) in indicated AXIN1-mutant HEK293T cell lines. Actin is used as loading control.

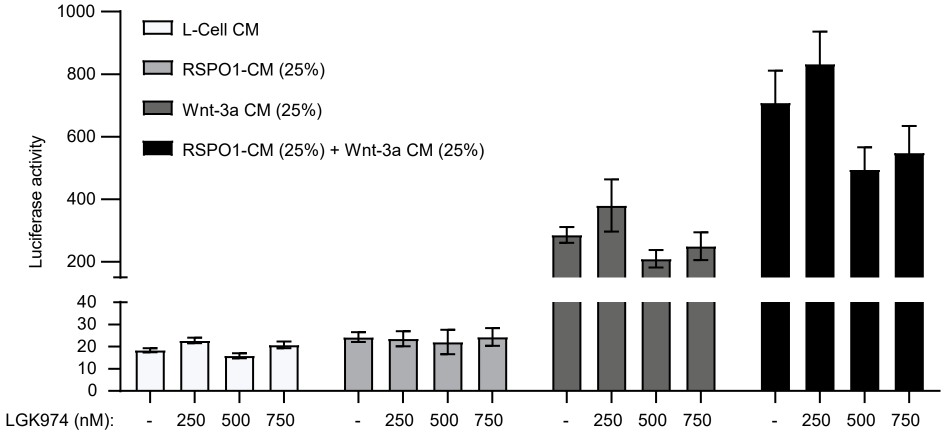

Supplemental figure S3. Related to figure 1 – Wnt/β-catenin pathway activity in Huh7 cells. TOPFlash reporter assay of Huh7 cells treated with Wnt-3a conditioned medium (CM), RSPO1 CM, or L-Cell CM as control. In addition, cells were treated with increasing concentrations of porcupine inhibitor LGK974. Graph shows a representative experiment (N=3) with mean +/- SD of N=2 wells.

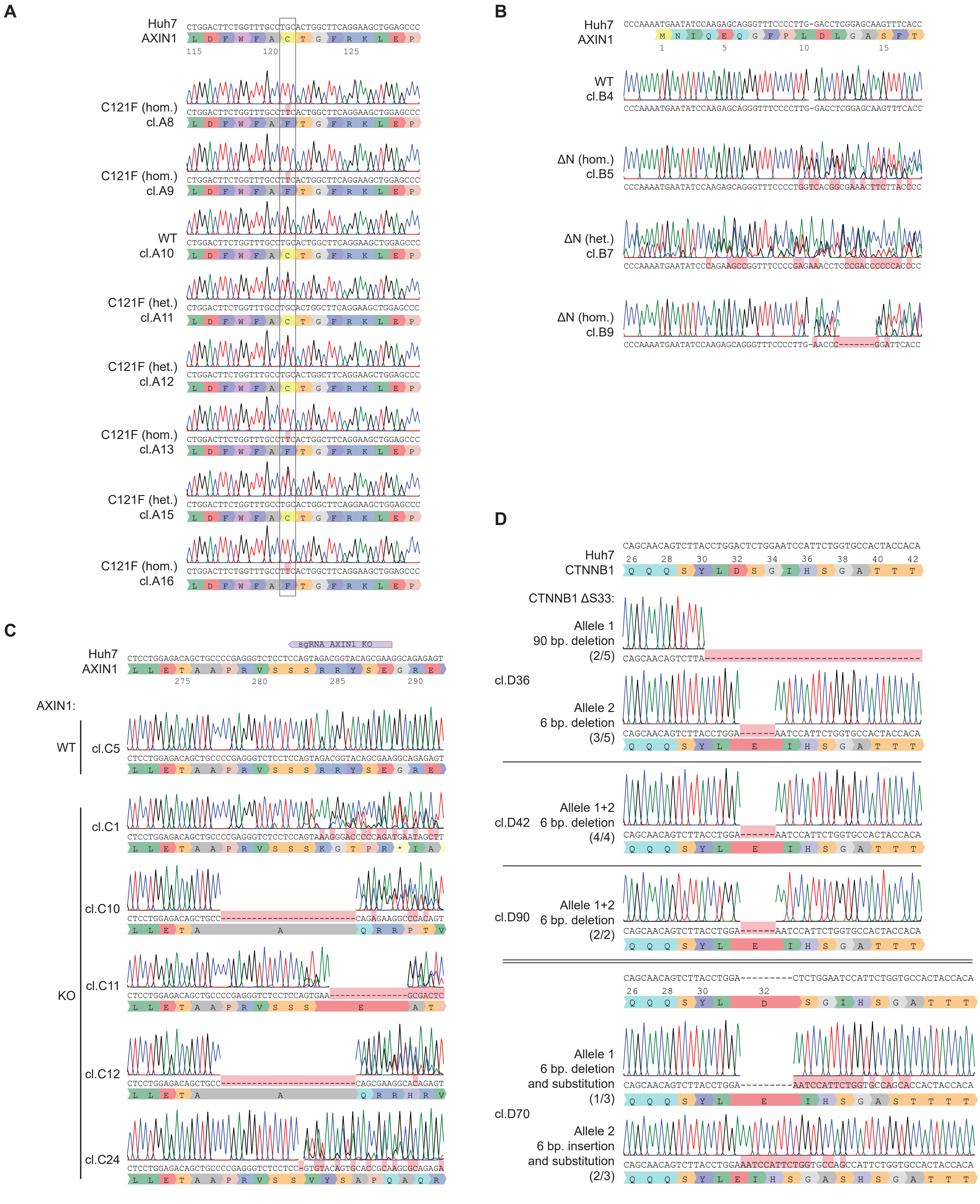

Supplemental figure S4. Related to figure 1, 2, 3 and 4 - Sanger sequencing of genetically modified Huh7 cells. Sanger sequencing results of Huh7 **(A)** AXIN1 C121F, **(B)** AXIN1 ΔN, **(C)** AXIN1 KO and **(D)** β-catenin ΔS33. For the β-catenin ΔS33 clones in (D), the frequency of each allele was determined by subcloning in pJET. Numbers between brackets indicate the amount of sanger sequencing reads found for each type of mutation.

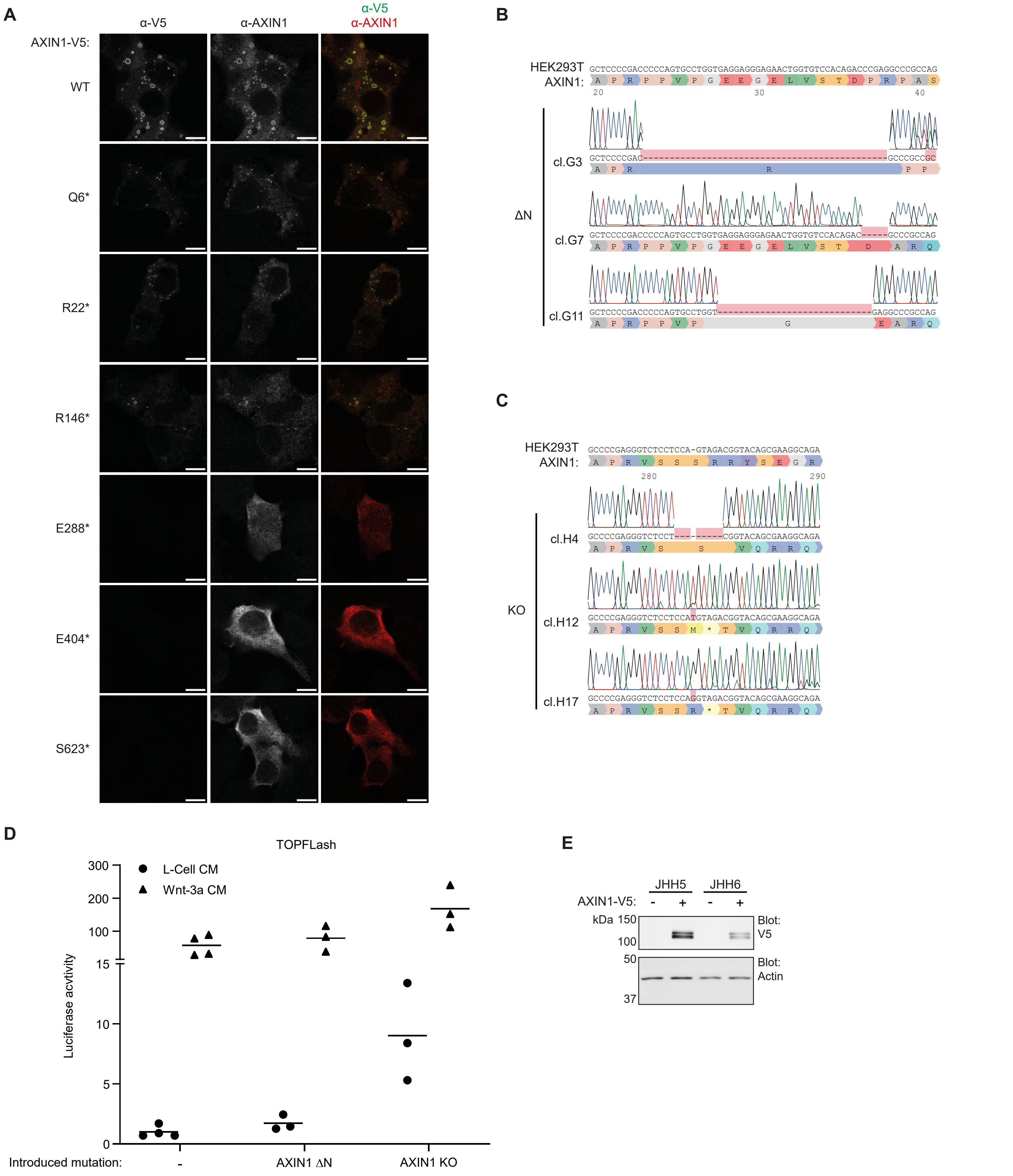

Supplemental figure S5. Related to figure 2 - Frameshift mutations in 5' coding regions yield an N-terminally truncated AXIN1 variant with partially retained functionality. (A) Representative immunofluorescence images of HEK293T cells overexpressing different AXIN1 truncating variants. Fixed cells were stained for V5 and AXIN1. Scale bars, 15 μm. **(B,C)** Sanger sequencing results of HEK293T cells harboring (B) AXIN1 ΔN and (C) AXIN1 KO. **(D)** TOPFlash reporter assay comparing non-modified HEK293T cells with clones harboring AXIN1 ΔN and KO mutations. Graph shows a representative experiment (n=3), where one dot represents the mean of technical duplicates of one clone. The horizontal line indicates the average of all tested clones with a similar genotype. Cells were treated with Wnt-3a conditioned medium (CM) or L-cell CM as control. **(E)** Western blot of JHH5 and JHH6 cells overexpressing AXIN-V5 for TOPFlash assay depicted in Figure 2K. Actin was used as a loading control.

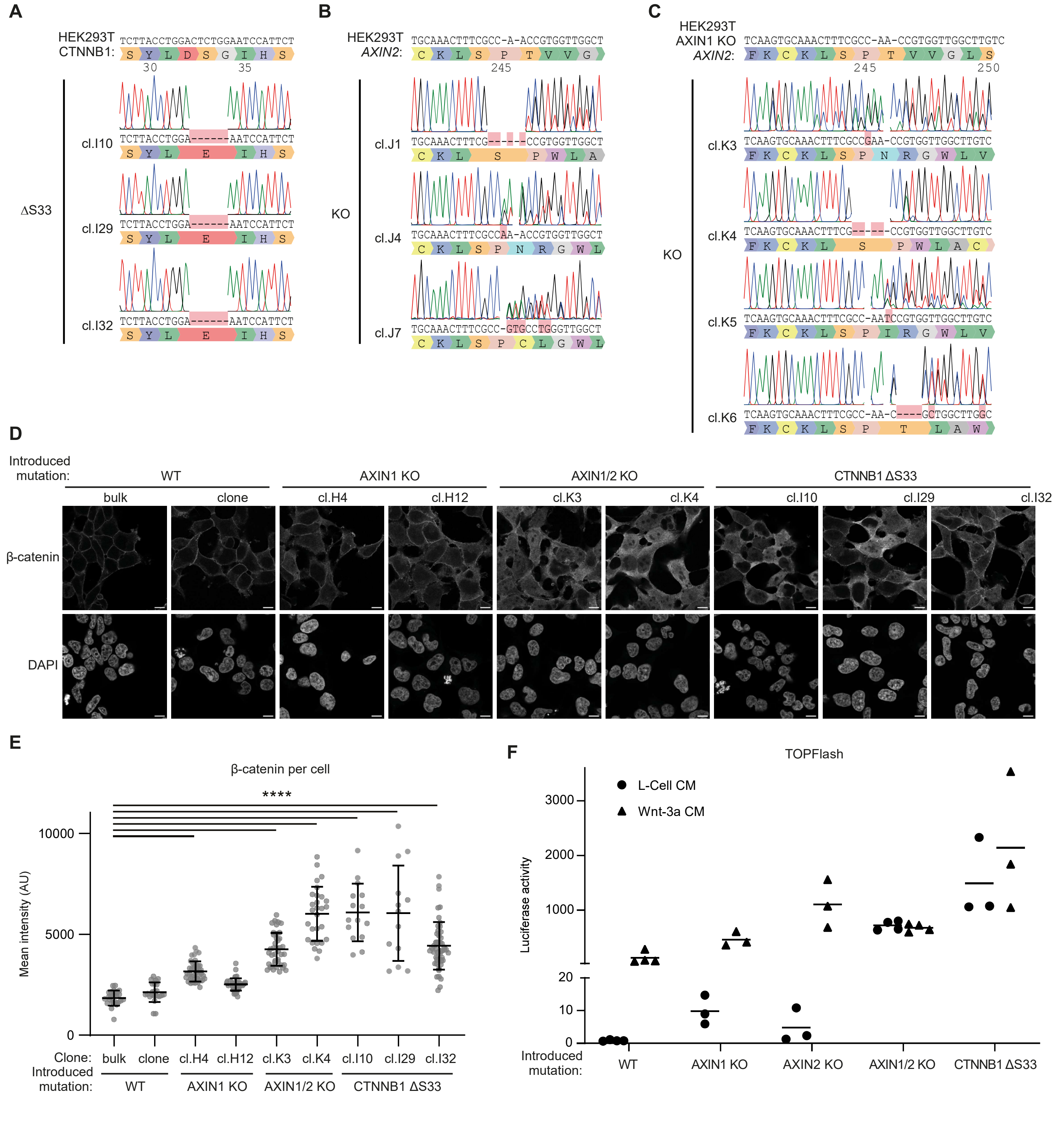

Supplemental figure S6. Related to figure 3 - AXIN1-mutant cells have moderate Wnt/β-catenin signaling levels, while CTNNB1-mutant cells are Wnt-high. Sanger sequencing results of HEK293T (A) β-catenin ΔS33, (B) AXIN2 KO, (C) as well as AXIN1 and AXIN2 KO. (D) Representative immunofluorescence images of HEK293T cells harboring different Wnt pathway mutations, labeled for β-catenin and DAPI. Scale bar represents 15 μm. (E) Quantification of immunofluorescence images from (D). Quantified n=272 cells using an automated ImageJ script as described in the methods section. Significance was determined using one-way ANOVA. **** indicates p ≤ 0.0001. (F) TOPFlash reporter assay comparing non-modified HEK293T cells to clones harboring different Wnt pathway mutations. Graph shows a representative experiment (n=3), where one dot represents the mean of technical duplicates of one clone. The horizontal line indicates the average of all tested clones with that genotype. Cells were treated with Wnt-3a conditioned medium (CM) or L-Cell CM as control.

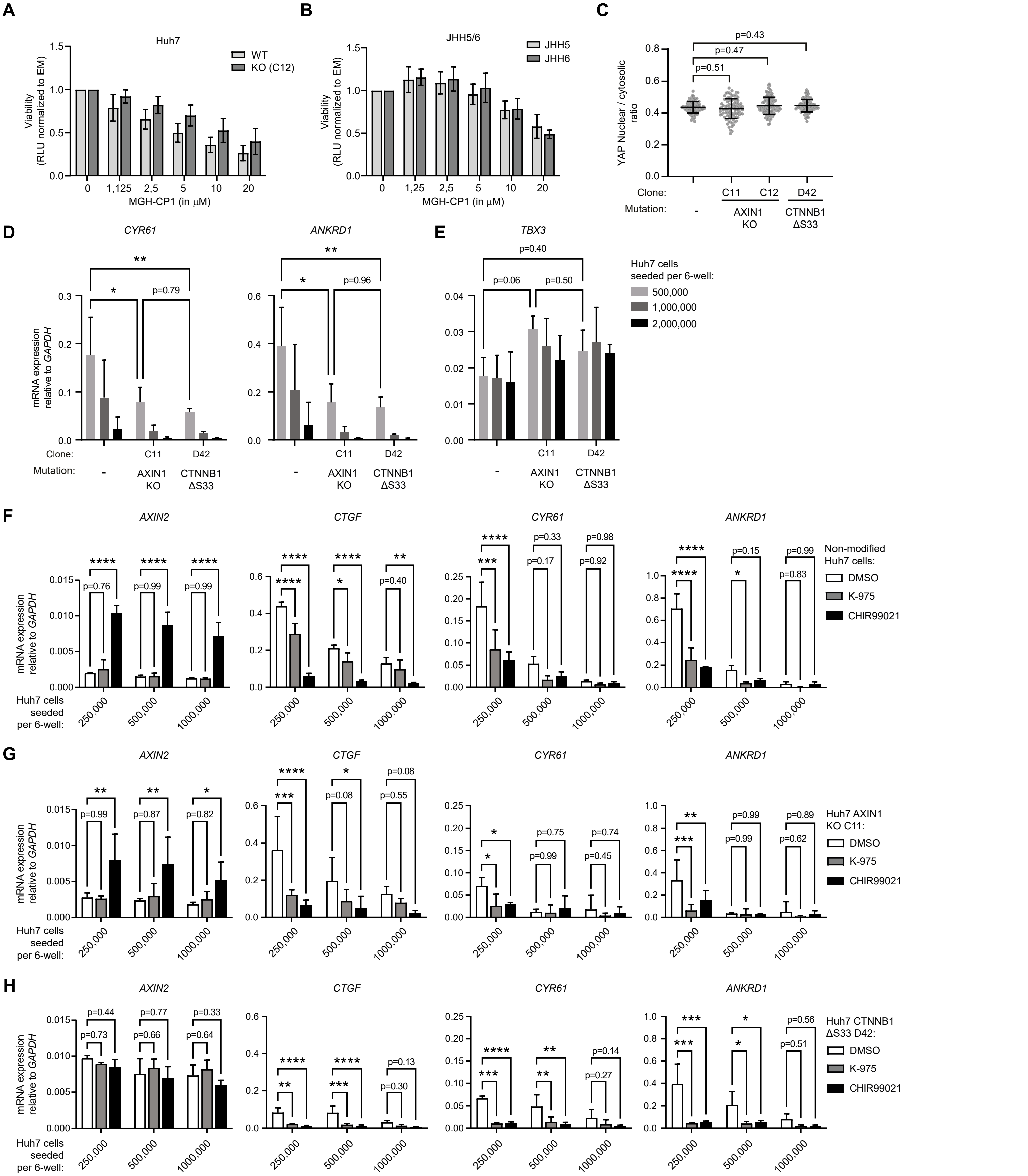

Supplemental figure S7. Related to figure 4 - Wnt pathway activation leads to dose-dependent inhibition of YAP/TAZ signaling in Huh7 cells. (A,B) Viability assays of (A) Huh7 WT and AXIN1 KO, and (B) JHH5 and JHH6 treated for five days with the indicated concentrations of MGH-C1. Bars and error bars represent the mean and standard deviation of respectively five and three biological replicates. **(C)** Quantification of immunofluorescence images of Fig. 4D. Quantified using an automated ImageJ script. Per cell, nuclear intensity levels were divided by the total cytosolic intensity to acquire a nuclear/cytosolic YAP ratio. One-way ANOVA was performed to determine significance. **(D,E)** RT-qPCR depicting expression of YAP/TAZ target genes (D) CYR61 and ANKRD1 and (E) TBX3 relative to the household gene GAPDH for different Huh7 clones at increasing cell density. Two-way ANOVA was performed to determine significance. **(F-H)** RT-qPCR for (F) non-modified, (G) AXIN1-deficient or (H) β-catenin-mutant Huh7 depicting expression of AXIN2, CTGF and CYR61 and ANKRD1 relative to the household gene GAPDH for Huh7 cells treated with the indicated small molecules at increasing cell density. Bars and error bars represent the mean and standard deviation of three biological replicates. Two-way ANOVA was performed to determine significance. * indicates $p \leq 0.05$, ** indicates $p \leq 0.01$, *** indicates $p \leq 0.001$, **** indicates $p \leq 0.0001$. Non-significant comparisons were left out for clarity.

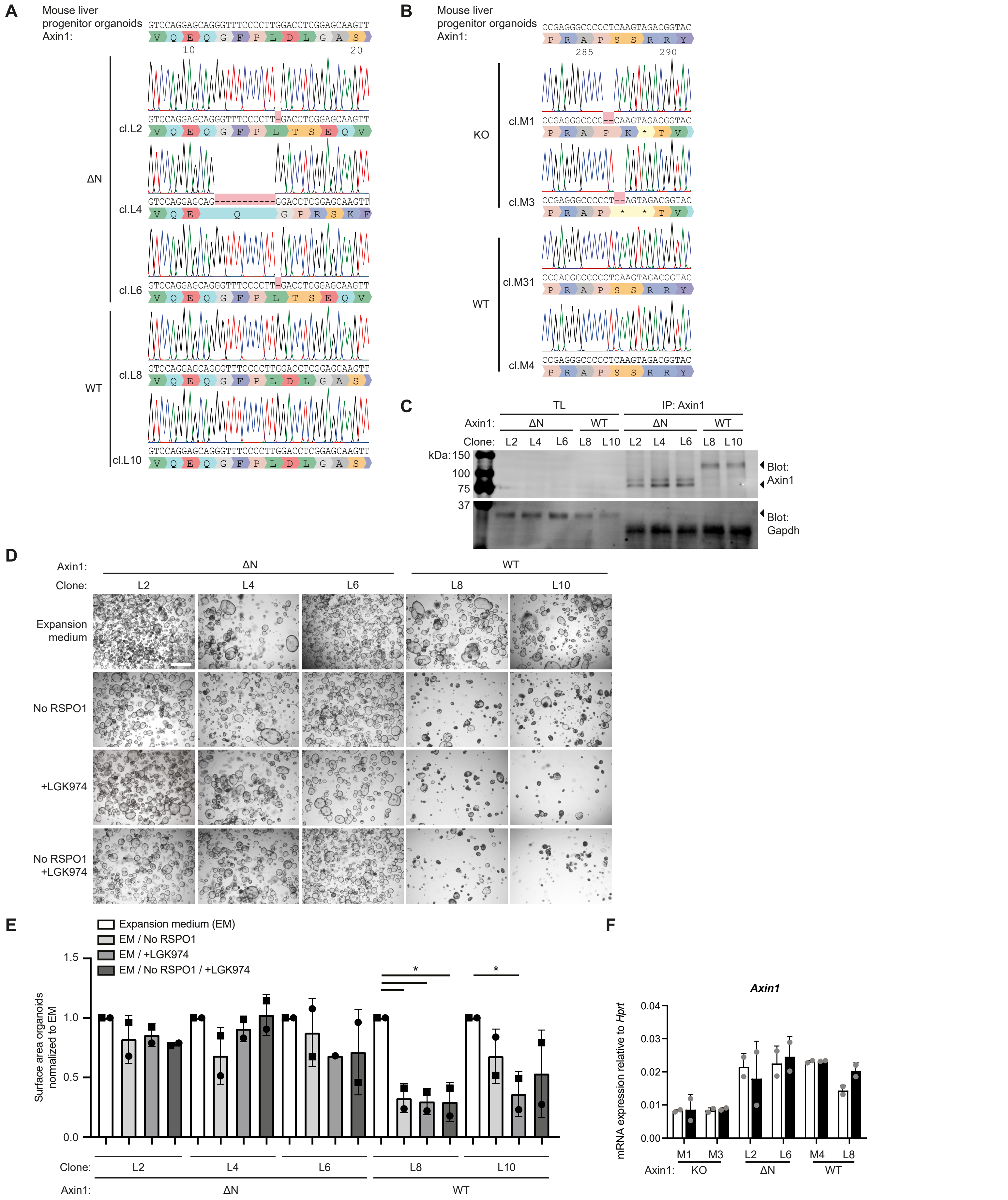

Supplemental figure S8. Related to figure 5 - Axin1 mutations promote Wnt ligand-independent growth in mouse liver progenitor organoids. (A,B) Sanger sequencing of mouse liver progenitor organoids harboring frameshifts leading to (A) Axin1 ΔN and (B) Axin1 KO. **(C)** Western blot of immunoprecipitated Axin1 from WT and Axin1 ΔN mouse liver progenitor organoids. Arrows indicate the WT and truncated Axin1 (detection with C76H11 for C-term Axin1). Gapdh was used as loading control, indicated by arrow. **(D)** Representative brightfield images of liver progenitor organoids cultured in expansion medium (EM) with and without 10% RSPO1 conditioned medium and 500 nM LGK974. See figure 5B for the protocol. The scale bar represents 500 μm. **(E)** Quantification of two biological replicates as performed in (D). Quantification was performed by determining cell surface area of the organoids using OrganoSeg analysis software[42]. Each condition was normalized to EM. Significance was determined using one-way ANOVA. * indicates $p \leq 0.05$. EM, Expansion medium. **(F)** RT-qPCR experiments for Axin1 mRNA levels relative to Hprt after treatment with 500 nM LGK974 or DMSO as control, similar to Figure 5E-G. Bars and error bars represent the mean of two biological replicates +/- SD. Given only n=2, the values for the biological replicates are depicted in grey.

Figure S9_Full blot figures

Full blots_Figure 1D

Blot:
V5

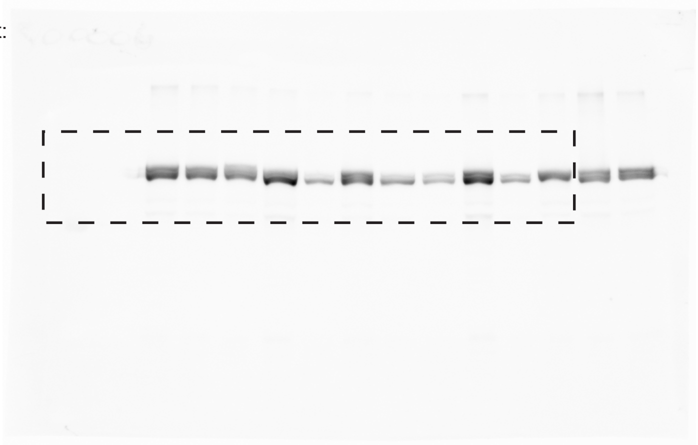

Blot:
V5

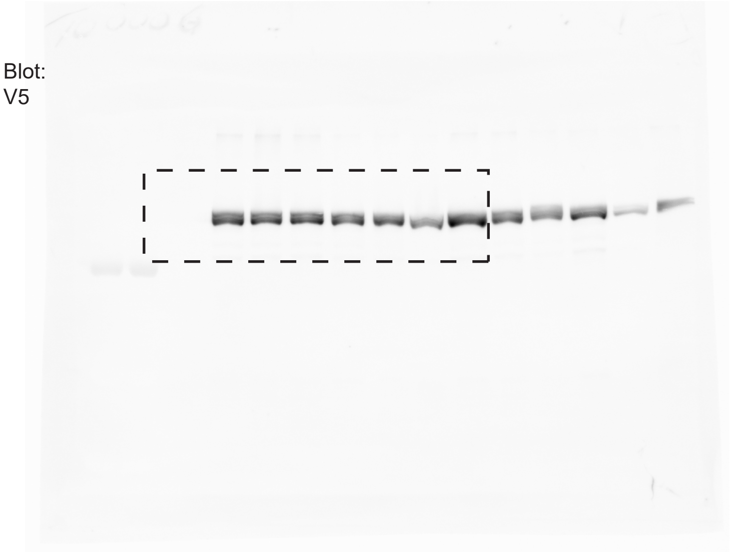

Blot:
Actin

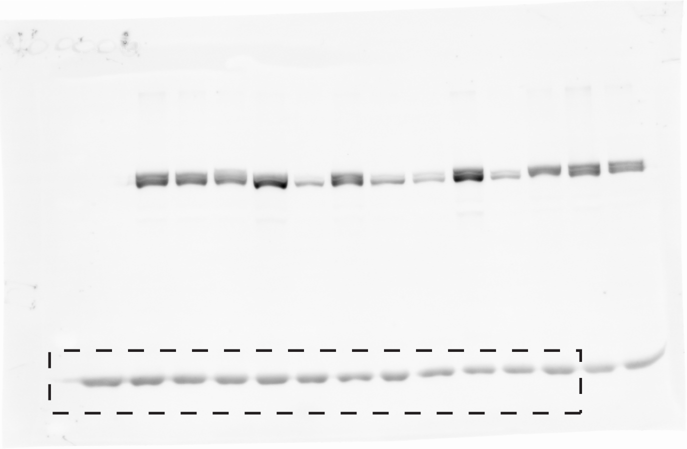

Blot:
Actin

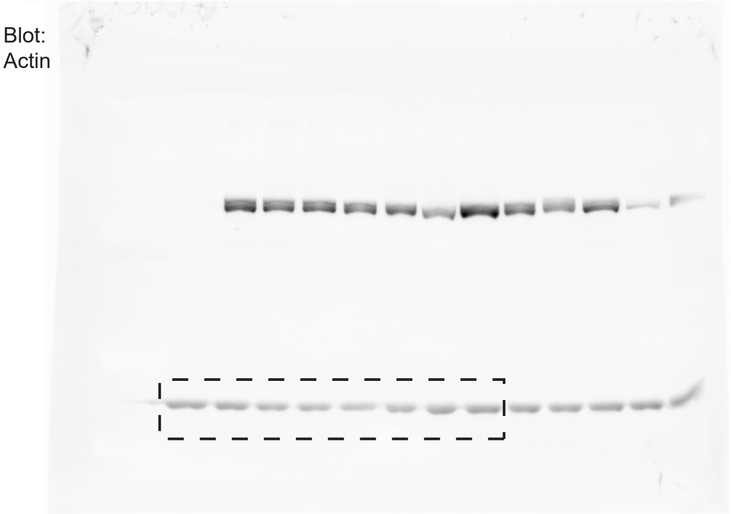

Full blots_Figure 2C

Blot:
AXIN1

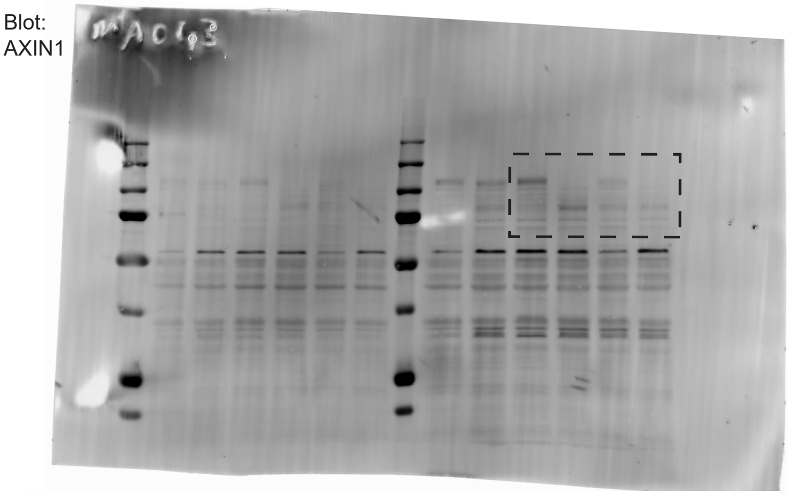

Blot:
Actin

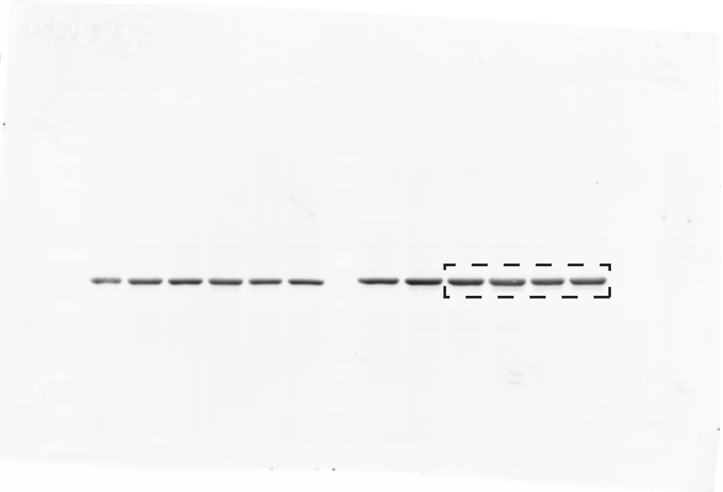

Full blots_Figure 2D

Blot:
AXIN1
C-term

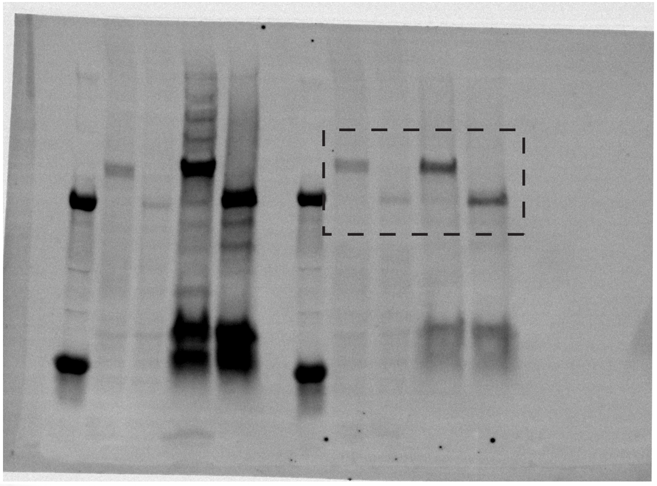

Blot:
GAPDH

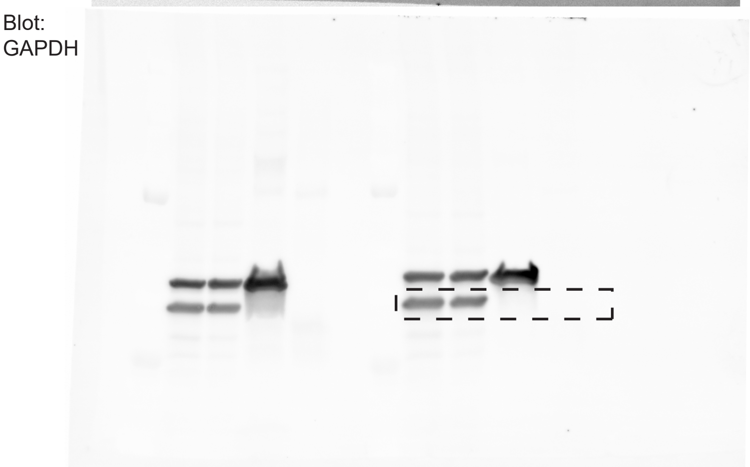

Full blots_Figure 2E

Blot:
V5

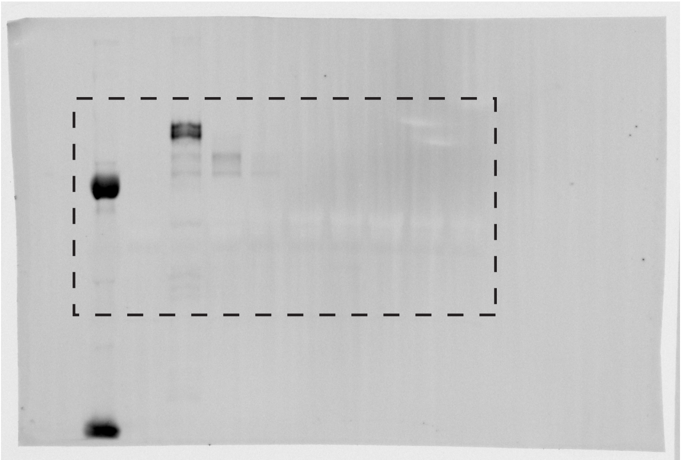

Blot:
Actin

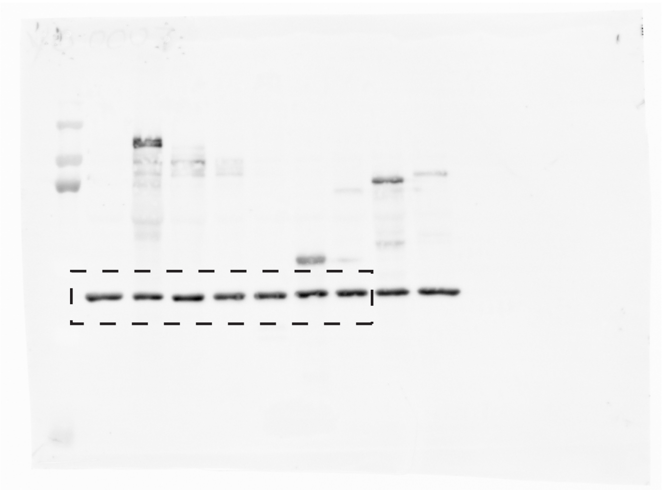

Blot:
AXIN1

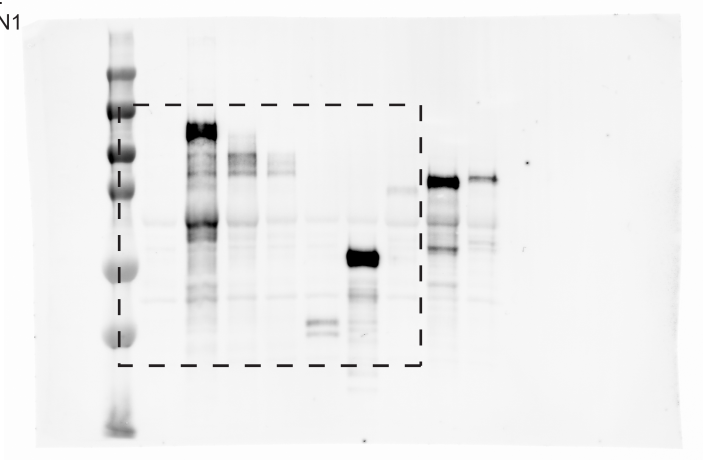

Full blots_Figure 2G

Blot:
V5

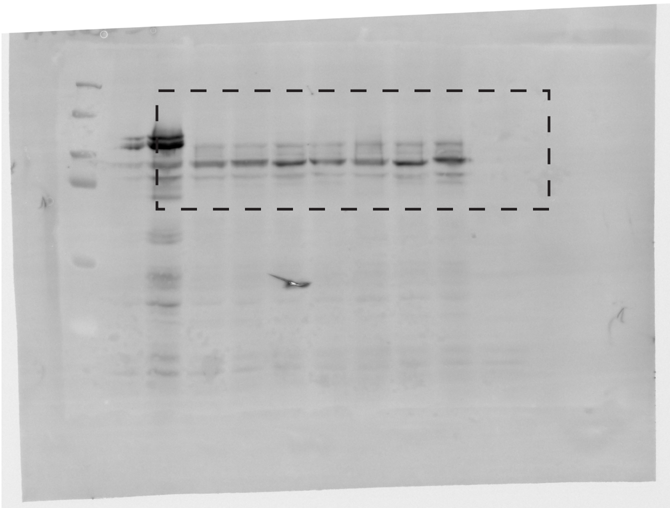

Blot:
Actin

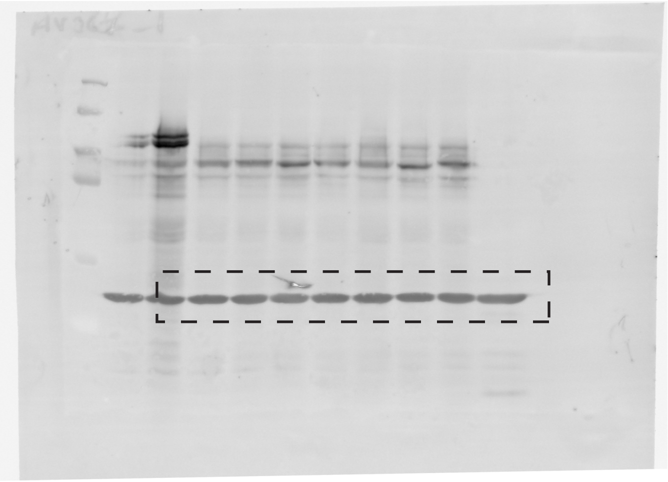

Full blots_Figure 2I

IP
Blot:
AXIN1

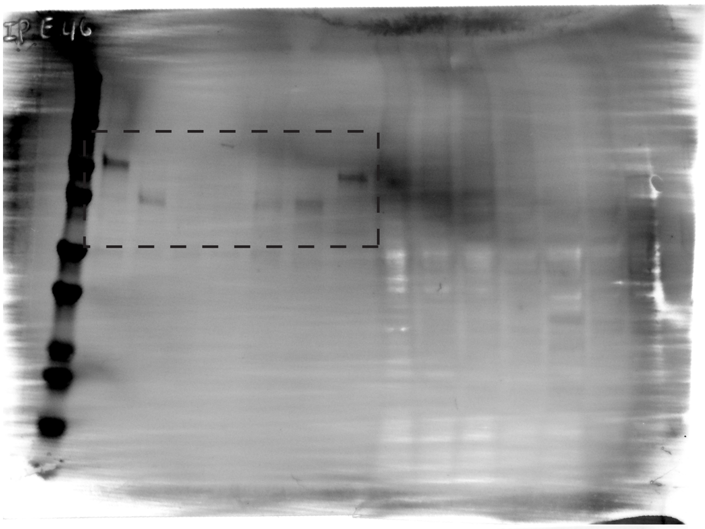

TL
Blot:
Actin

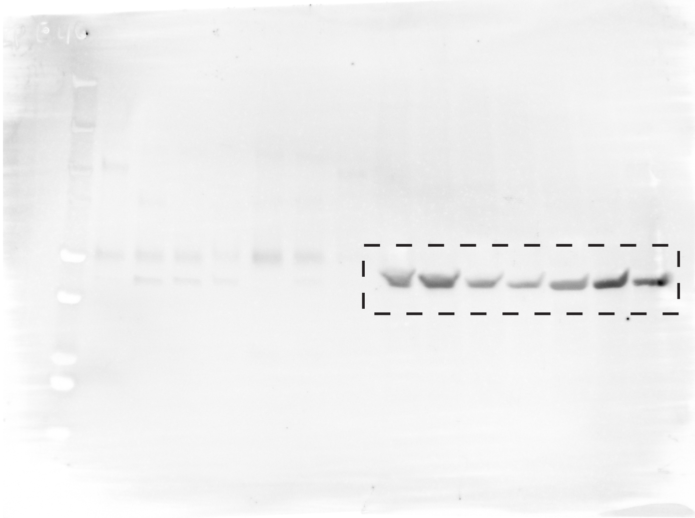

Full blots_Figure 5A

Blot:
Axin1

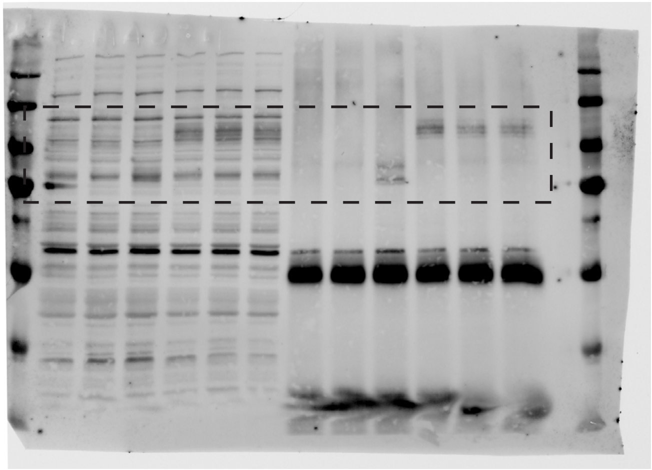

Blot:
Actin

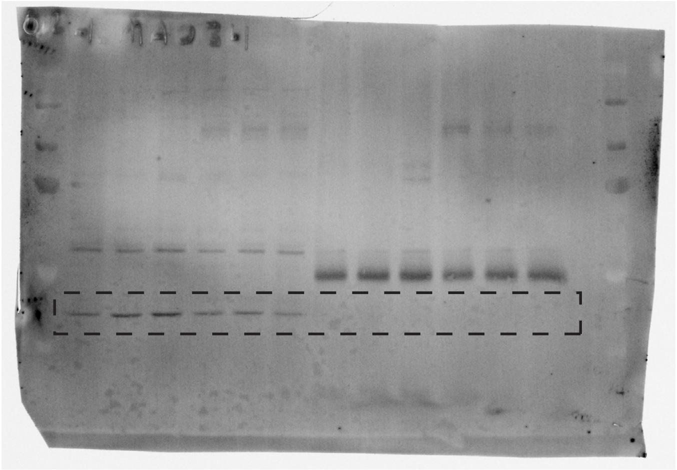

Full blots_Figure S2F

Blot:
APC

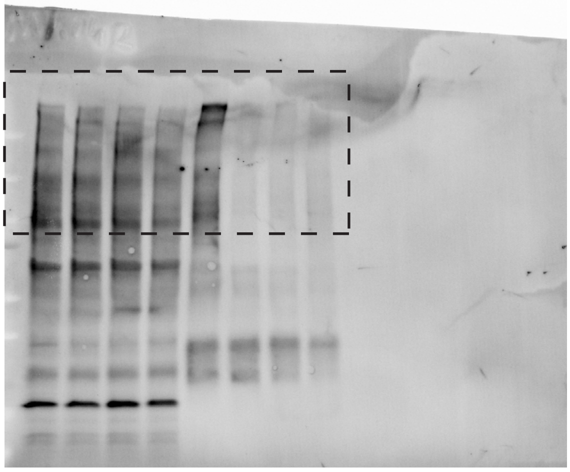

Blot:
AXIN1

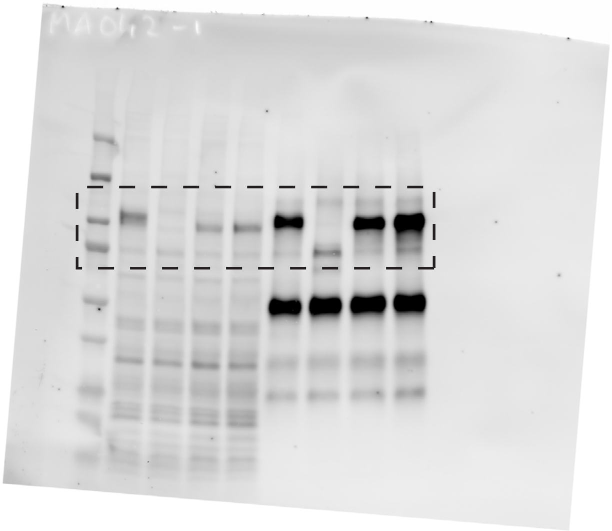

Blot:
β-catenin

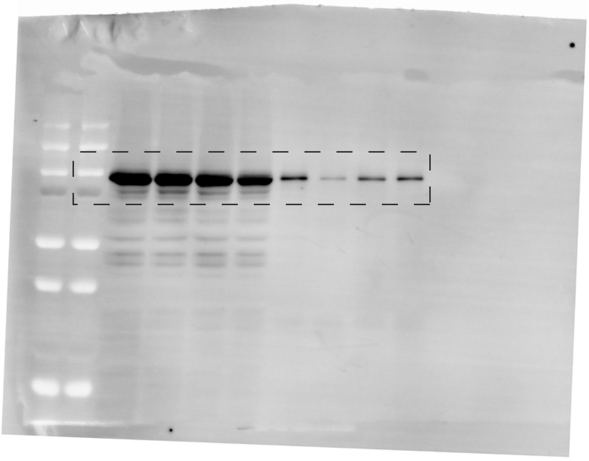

Blot:
GSK3

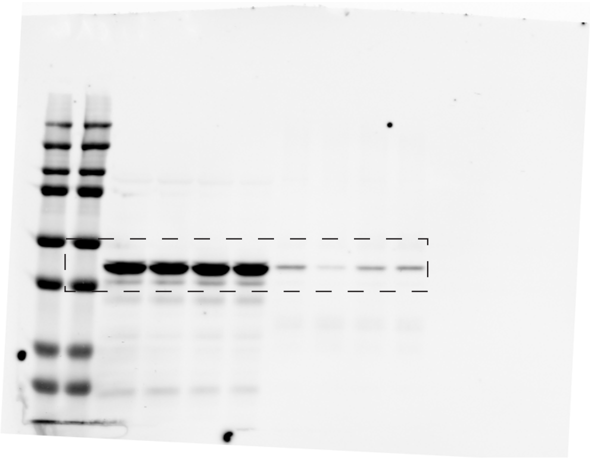

Blot:
Actin

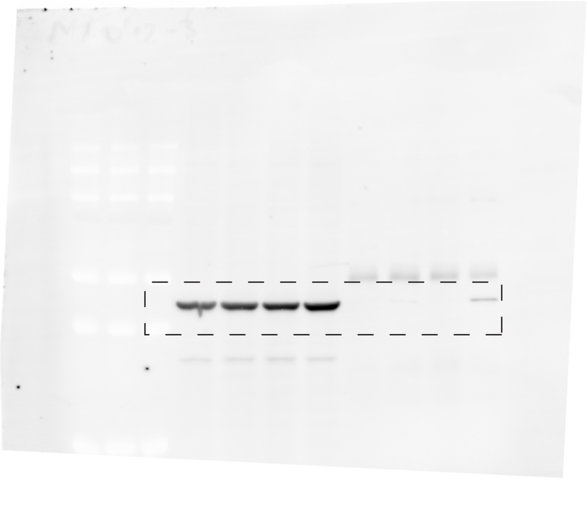

Full blots_Figure S5E

Blot:
V5

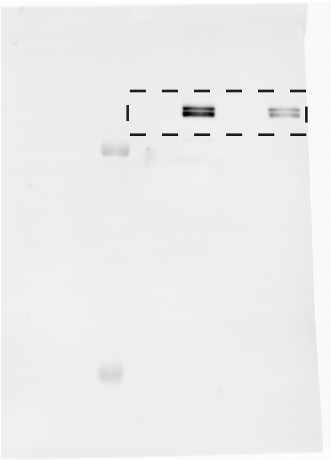

Blot:
Actin

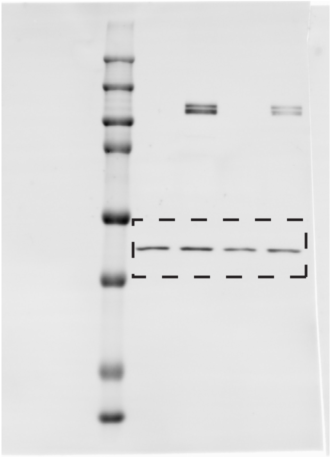

Full blots_Figure S8C

Blot:
Axin1

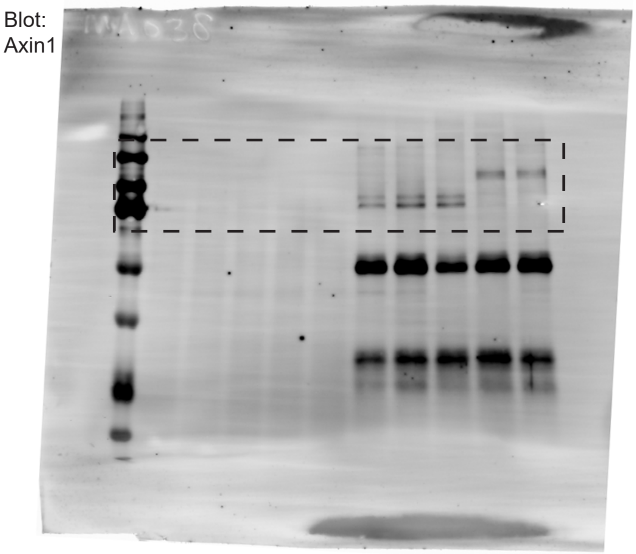

Blot:
Gapdh

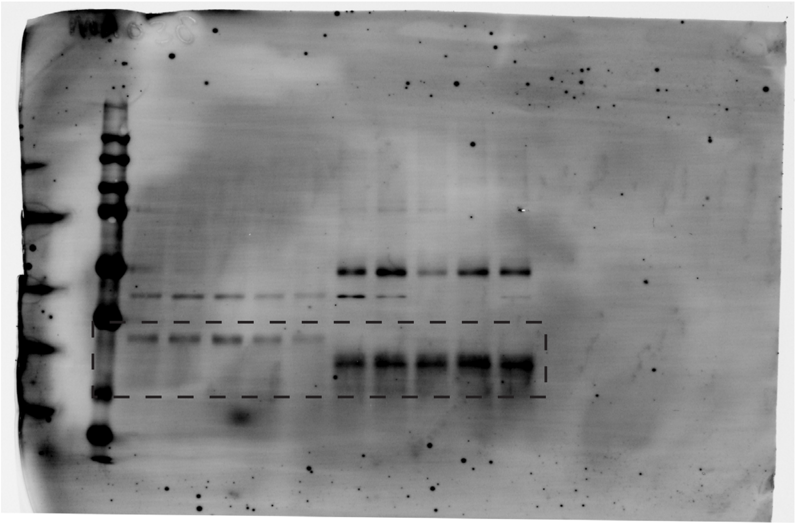

Table S1

**CRISPR/Cas9 oligos (including overhang)**	
sgRNA h*AXIN1* 5' exon 2 fw	caccgAACTTGCTCCGAGGTCCAAG
sgRNA h*AXIN1* 5' exon 2 rv	aaacCTTGGACCTCGGAGCAAGTT
sgRNA h*AXIN1* 3' exon 2 fw	caccgTTCGCTGTACCGTCTACTGG
sgRNA h*AXIN1* 3' exon 2 rv	aaacCCAGTAGACGGTACAGCGAAC
sgRNA hAXIN1 L106R fw	caccGGTTCAGGACTTTCCTGAAGC
sgRNA hAXIN1 L106R rv	aaacGCTTCAGGAAAGTCCTGAACC
ssDNA for hAXIN1 L106R knock-in (* denotes phosphorothioate bonds)	*T*CTTCTCCTCGTTCGAGTCACAGGGCTCCAGCTTCCTGAAGCCAGTGCAGGCAAACCAGAAGTCCAGCAAGTCGGCACAGCCCTCTTGCTTTCGAAAAGTCCTGAACAGGCTTATCCCATCTT*G*G
sgRNA h*AXIN2* 3' exon 2 fw	caccGTGCAAACTTTCGCCAACCG
sgRNA h*AXIN2* 3' exon 2 rv	aaacCGGTTGGCGAAAGTTTGCAC
sgRNA m*Axin1*1 5' exon 2 fw	caccGCTTTCCCAGAACAGAAACTG
sgRNA m*Axin1*1 5' exon 2 rv	aaacCAGTTTCTGTTCTGGGAAAGC
sgRNA m*Axin1*1 3' exon 2 fw	caccGTTGTACCGTCTACTTGAGG
sgRNA m*Axin1*1 3' exon 2 rv	aaacCCTCAAGTAGACGGTACAAC
**Prime editing oligos (including overhang)**	
hCTNNB1 ΔS33 pegRNA spacer (excluding overhangs), designed by Sabine Fuchs lab[35]	CAACAGTCTTACCTGGACTC
hCTNNB1 ΔS33 3' extension sequence (excluding overhangs), designed by Sabine Fuchs lab[35]	TGGCACCAGAATGGATTTCCAGGTAAGAC
hCTNNB1 ΔS33 Nicking sgRNA (PE3) (excluding overhangs), designed by Sabine Fuchs lab[35]	CCACTCATACAGGACTTGGG
h*AXIN1* C121F pegRNA spacer fw	caccGCAGCTTCCTGAAGCCAGTGCgtttt
h*AXIN1* C121F pegRNA spacer rv	ctctaaaacGCACTGGCTTCAGGAAGCTGC
pegRNA scaffold fw	AGAGCTAGAAATAGCAAGTTAAAATAAGGCTAGTCCGTTATCAACTTGAAAAAGTGGCACCGAGTCG
pegRNA scaffold rv	GCACCGACTCGGTGCCACTTTTTCAAGTTGATAACGGACTAGCCTTATTTTAACTTGCTATTTCTAG
hAXIN1 C121F pegRNA extension fw	gtgcTGGTTTGCCTtCACTGGCTTCAGGAAG
hAXIN1 C121F pegRNA extension rv	aaaaCTTCCTGAAGCCAGTGaAGGCAAACCA
hAXIN1 C121F Nicking sgRNA (PE3b) fw	caccGACTTCTGGTTTGCCTtCAC
hAXIN1 C121F Nicking sgRNA (PE3b) rv	aaacGTGaAGGCAAACCAGAAGTC
**Genotyping primers**	
gPCR h*AXIN1* 5' exon 2 fw	GCGTCATCGTGAGTCTTGTC

gPCR h*AXIN1* 5' exon 2 rv	TGTCTCCAGGAGCAGCTT
gPCR h*AXIN1* 5' exon 2 fw	CCCCCACCCCACCATACTTGAA
gPCR h*AXIN1* 5' exon 2 rv	TCATCAGCACCTTTCCCTGGCT
gPCR h*AXIN2* 5' exon 2 fw	GCTGAAGCCTGCCACCAAGACC
gPCR h*AXIN2* 5' exon 2 rv	CCACAACCCAGCTGCCTCCCTA
hCTNNB1 ΔS33 fw	GCCTGGATGCAGTACCATTCTTCCAC
hCTNNB1 ΔS33 rv	AGACACCATCTGAGGAGAACGCA
hCTNNB1 ΔS33 sequencing primer	ACACTCACTATCCACAGTTCAGCA
gPCR m*Axin1* 5' exon 2 fw	AGATGTCCTCCATGACTCAGGCT
gPCR m*Axin1* 5' exon 2 rv	ACCCTGAGCTCTGGTCACTGCA
gPCR m*Axin1* 3' exon 2 fw	AGCCACCCCAAGACGTTCAGAT
gPCR m*Axin1* 3' exon 2 rv	TCCTTGCTCCTTTGCCAGGTCT
**qRT-PCR primers**	
h*GAPDH* fw	CTTTTGCGTCGCCAG
h*GAPDH* rv	TTGATGGCAACAATATCCAC
h*AXIN1* fw	CCTGTGGTCTACCCGTGTCT
h*AXIN1* rv	GCTATGAGGAGTGGTCCAGG
h*AXIN2* fw	AAAGAGAGGAGGTTCAGATG
h*AXIN2* rv	CTGAGTCTGGGAATTTTTCTTC
h*LGR5* fw	GGTGACAACAGCAGTATGGACGA
h*LGR5* rv	GAAGGTGAACACTGCACTGAATGAA
h*CYR61* fw	GATCTGCAGAGCTCAGTCAGAG
h*CYR61* rv	CCATCAATACATGTGCACTG
h*CTGF* fw	CTGGAAGAGAACATTAAGAAGG
h*CTGF* rv	GGTATGTCTTCATGCTGGTG
h*AREG* fw	GAGCCGACTATGACTACTCAGA
h*AREG* rv	TCACTTTCCGTCTTGTTTTGGG
h*ANRKD1* fw	AGTAGAGGAACTGGTCACTGG
h*ANRKD1* rv	TGTTTCTCGCTTTTCCACTGTT
h*TBX3* fw	AGCGATCACGCAACGTGGCA
h*TBX3* rv	GGCTTCGCTGGGACACAGATCTTT
m*Hprt* fw	AAGCTTGCTGGTGAAAAGGA
m*Hprt* rv	TTGCGCTCATCTTAGGCTTT
m*Axin1* fw	ACCCAGTACCACAGAGGACG
m*Axin1* rv	CTGCTTCCTCAACCCAGAAG
m*Axin2* fw	GGACTGGGGAGCCTAAAGGT
m*Axin2* rv	AAGGAGGGACTCCATCTACGC
m*Lgr5* fw	AGAACACTGACTTTGAATGG
m*Lgr5* rv	CACTTGGAGATTAGGTAACTG
m*Alb* fw	GCGCAGATGACAGGGCGGAA

m*Alb* rv	GTGCCGTAGCATGCGGGAGG
m*Cyp4f14* fw	TGCTGCTGAGCAAGGATGAA
m*Cyp4f14* rv	TGCCTTGCCAGGTTGTAGAG
m*Hnf4a* fw	GCTAAGGCGTGGGTAGGG
m*Hnf4a* rv	AGGCTGTTGGATGAATTGAGG
m*Cyp2c9* fw	TAATGCATTCCCGATACTCTTGCGT
m*Cyp2c9* rv	TCTCAGGATTCCCTTTGGCCTTC

Method S1 – Macro ImageJ analysis

```
DAPI = "1"
bCat = "2"
title = getTitle();
run("Duplicate...", "duplicate channels=" + DAPI);
DAPIdup = getTitle();
run("Gaussian Blur...", "sigma=10");
setAutoThreshold("Default dark");
setOption("BlackBackground", false);
run("Convert to Mask");
selectImage(title);
run("Select None");
run("Duplicate...", "duplicate channels=" + bCat);
bCatdup = getTitle();
run("Median...", "radius=20");
setAutoThreshold("Minimum dark");
waitForUser("ok")
run("Convert to Mask");
selectImage(title);
run("Select None");
run("Duplicate...", "duplicate channels=" + DAPI);
run("Gaussian Blur...", "sigma=20");
run("Find Maxima...", "prominence=20 output=[Segmented Particles]");
segmentdup = getTitle();
imageCalculator("Min create", bCatdup,segmentdup);
outlinedup = getTitle();
run("Analyze Particles...", "clear add");
imageCalculator("Min create", bCatdup,segmentdup);
selectImage(DAPIdup);
run("Select None");
run("Invert");
imageCalculator("Min create", outlinedup, DAPIdup);
cytodup = getTitle();
roiManager("Show All");
waitForUser("please check valid cytosols");
roiManager("Show None");
run("Select None");
nROIs = roiManager("count");
for (i = 0; i < nROIs; i++) {
	selectImage(cytodup);
	run("Duplicate...", " ");
	run("Invert");
	roiManager("Select", i);
	run("Clear Outside");
```

```
		run("Select None");
		run("Invert");
		run("Convert to Mask");
		run("Create Selection");
		selectImage(title);
		Stack.setChannel(bCat);
		run("Restore Selection");
		//waitForUser("OK");
		run("Measure");
		bottomrow = (nResults-1);
		print(bottomrow);
		area = getResult("Area", bottomrow);
		print(area);
		//waitForUser("OK");
		if (area<100){
			IJ.deleteRows(bottomrow, bottomrow);
		} else if (area>1000){
			IJ.deleteRows(bottomrow, bottomrow);
		}
		//waitForUser("OK");
		run("Select None");
		selectImage(DAPIdup);
		run("Select None");
		run("Duplicate...", " ");
		roiManager("Select", i);
		run("Clear Outside");
		run("Select None");
		run("Invert");
		run("Convert to Mask");
		run("Create Selection");
		//waitForUser("OK");
		selectImage(title);
		Stack.setChannel(bCat);
		run("Restore Selection");
		run("Measure");
		bottomrow = (nResults-1);
		if (area<100){
			IJ.deleteRows(bottomrow, bottomrow);
		} else if (area>1000){
			IJ.deleteRows(bottomrow, bottomrow);
		}
		//waitForUser("OK");
	}
	waitForUser("please check cytosoldata");
```
